# Forecasting adverse surgical events using self-supervised transfer learning for physiological signals

**DOI:** 10.1038/s41746-021-00536-y

**Published:** 2021-12-08

**Authors:** Hugh Chen, Scott M. Lundberg, Gabriel Erion, Jerry H. Kim, Su-In Lee

**Affiliations:** 1 Paul G. Allen School of Computer Science and Engineering, University of Washington, 185 E Stevens Way NE, Seattle, WA 98195 USA; 2 Microsoft Research, 14820 NE 36th St, Redmond, WA 98052 USA; 3 Medical Scientist Training Program, University of Washington, 1959 NE Pacific St, Seattle, WA 98195 USA; 4 Global Innovation Exchange, University of Washington, 12280 NE District Wy, Bellevue, WA 98005 USA

**Keywords:** Hypoxia, Risk factors

## Abstract

Hundreds of millions of surgical procedures take place annually across the world, which generate a prevalent type of electronic health record (EHR) data comprising time series physiological signals. Here, we present a transferable embedding method (i.e., a method to transform time series signals into input features for predictive machine learning models) named PHASE (PHysiologicAl Signal Embeddings) that enables us to more accurately forecast adverse surgical outcomes based on physiological signals. We evaluate PHASE on minute-by-minute EHR data of more than 50,000 surgeries from two operating room (OR) datasets and patient stays in an intensive care unit (ICU) dataset. PHASE outperforms other state-of-the-art approaches, such as long-short term memory networks trained on raw data and gradient boosted trees trained on handcrafted features, in predicting six distinct outcomes: hypoxemia, hypocapnia, hypotension, hypertension, phenylephrine, and epinephrine. In a transfer learning setting where we train embedding models in one dataset then embed signals and predict adverse events in unseen data, PHASE achieves significantly higher prediction accuracy at lower computational cost compared to conventional approaches. Finally, given the importance of understanding models in clinical applications we demonstrate that PHASE is explainable and validate our predictive models using local feature attribution methods.

## Introduction

Globally, the number of surgical operations performed each year exceeds 300 million [1]. Although surgeries are crucial components of medical care, they have a high prevalence of adverse events (i.e., patients harmed as a result of their medical treatment) relative to other medical specialties (46–65% of all adverse events are surgery-related [2]). In fact, several international studies have shown rates of adverse events ranging from 3 to 22% in surgical patients [3–5]. Fortunately, these studies also conclude that the majority of adverse events are preventable, indicating a tremendous opportunity for improvement by predictive models.

The accuracy of such models is largely dependent on the availability of training data. As of 2014, a large portion (>40%) of invasive, therapeutic surgeries take place in hospitals with either medium or small numbers of beds [6, 7]. These smaller institutions may lack either sufficient data or computational resources to train accurate models. Furthermore, patient privacy considerations mean that large public EHR datasets are unlikely, leaving many institutions with insufficient resources to train performant models on their own. In the face of this insufficiency, one natural way to make accurate predictions is *transfer learning*, which has already shown success in medical images as well as clinical text [8–10]. Particularly with the popularization of wearable sensors for health monitoring [11], transfer learning techniques that train models in one dataset and use them in another are arguably underexplored for physiological signals, which account for a significant portion of the hundreds of petabytes of currently available worldwide health data [12, 13]. One promising avenue of transfer learning research is *deep embedding models* which learn to extract generalizable features from images or time-series data [14, 15] which improve over traditional domain-specific hand engineered features.

Our approach, PHASE (PHysiologicAl Signal Embeddings), trains deep embedding models on physiological signals to better forecast and facilitate prevention of potentially millions of adverse surgical outcomes. Furthermore, these models not only improve predictive accuracy but can be transferred from an institution with plentiful computational resources to institutions with less. PHASE improves over previous approaches in two important ways:

- PHASE *improves predictive accuracy* by leveraging deep learning to embed physiological signals. Using long-short term memory networks (LSTMs), PHASE embeds physiological signals prior to forecasting adverse events with a downstream model. We investigate a number of self-supervised approaches (training with inputs and outputs derived from the signal data itself) [16] to effectively train embedding models. Our results show that gradient boosted tree (GBT) models trained with features extracted by self-supervised LSTMs improves accuracy over conventional approaches for forecasting surgical outcomes that rely on a single model (i.e., predicting adverse outcomes with an LSTM with raw features or a GBT with raw or hand engineered features).
- PHASE *shares models rather than data* to address data insufficiency and improves over alternative methods including GBTs trained with raw features, hand engineered features, and embeddings jointly learned by a single LSTM. Data insufficiency is especially important for surgical data because protecting patient privacy makes it difficult to share large amounts of medical data which exacerbates the lack of publicly available data [17]. By transferring performant models as has been done in medical images and clinical text [8–10], scientists can collaborate to improve accuracy of predictive models without exposing patient data.

**Fig. 1:**
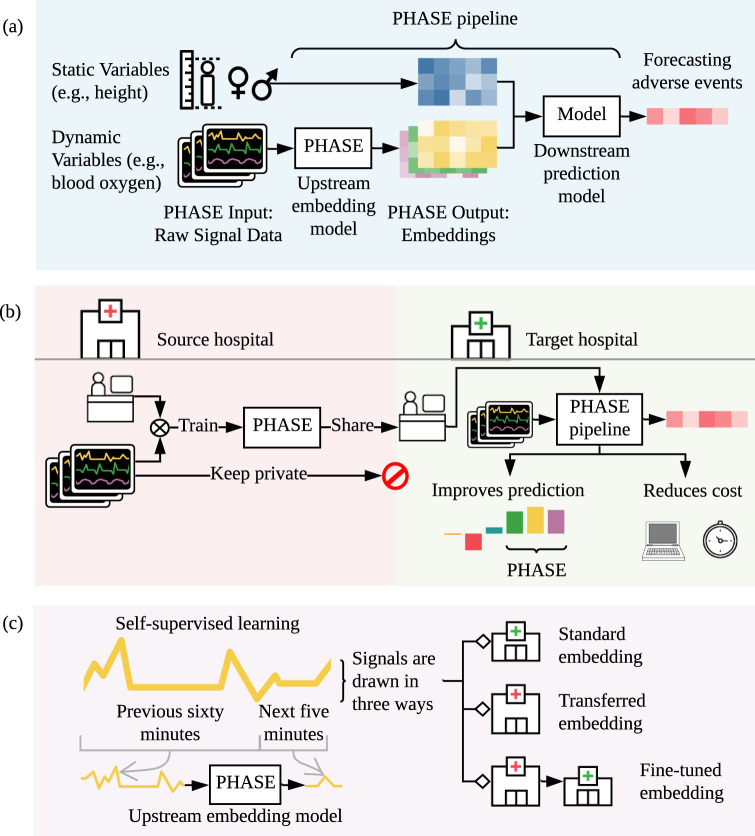
The high-level goal of PHASE. **a** PHASE learns models that embed (i.e., extract features from) physiological signals. We concatenate these embeddings with static data to predict adverse events. We describe the model extracting features as an *upstream embedding model* and the model making the final prediction as the *downstream prediction model*. **b** PHASE enables researchers at different hospitals to work together without sharing data. Researchers can perform transfer learning where upstream embedding models are trained on data drawn from a *source hospital* and used to embed signals and make a downstream prediction in data drawn from a *target hospital*. We show that this approach outperforms conventional deep learning and tree models trained with raw or hand engineered features. In addition, this approach reduces computational cost for users in target hospitals. **c** PHASE comprises LSTM embedding models trained per physiological signal that predict the future of the signal based on the past (self-supervised learning). We train self-supervised embedding models using data drawn in three distinct ways: (1) from the target hospital (standard embedding), (2) from a distinct source hospital (transferred embedding), and (3) from a distinct source hospital and then the target hospital (fine-tuned embedding) (More details in Results section “Overview of the PHASE framework”).

In contrast to prior research on transfer learning for physiological signals that focus on a single medical center’s electroencephalograms (EEGs) [18] or intensive care unit (ICU) stays [19], we evaluate transfer learning across three distinct medical center datasets (two from operating rooms and one from an ICU). Furthermore, we focus on evaluating self-supervised approaches (Fig. 1) to train embedding models that we validate with feature attributions. To achieve this, we use data collected by the Anesthesia Information Management System (AIMS) from two medical centers as well as the Medical Information Mart for Intensive Care (MIMIC-III) dataset [20]. We utilize fifteen physiological signal variables and six static variable inputs (variables listed in Results section “Five perioperative outcomes from three hospital datasets”) to forecast six possible outcomes: hypoxemia, hypocapnia, hypotension, hypertension, phenylephrine administration, and epinephrine administration. We show in a standard embedding setting, PHASE outperforms a number of conventional approaches across six outcomes of interest: hypoxemia, hypocapnia, hypotension, hypertension, phenylephrine administration, and epinephrine administration. Our results suggest that if the previous state of the art machine learning model (a gradient boosted tree model using hand engineered features [21]) captured 15% of hypoxemic events, PHASE captures approximately 19% of hypoxemic events based on a fixed precision. Although 19% of events may seem low, PHASE stands to benefit practitioners in two ways: (1) offloading mental burden from practitioners who are not trained to forecast adverse events and (2) a higher detection rate than that of practicing anesthesiologists (who were outperformed by the previous state of the art [21]). Quantitatively speaking, we observe ~2.3 hypoxemic events per surgery in our data, in the US alone our method could forecast roughly 5 million hypoxemic events that the previous state of the art model fails to capture (given that there are an estimated 50 million surgeries in the US annually [22]).

Furthermore, we show that PHASE improves performance in a transferred embedding setting where LSTM embedding models are trained in one dataset and used to extract features in a completely unseen dataset. Building upon this finding, we show that fine-tuning the LSTMs on unseen data leads to faster convergence and improved predictive performance compared to randomly initialized models across all outcomes. Finally, we validate our models by identifying important variables using state of the art local feature attribution methods [23]. We interpret our models to validate that the models uncover statistical patterns that agree with prior literature and demonstrate that models trained using PHASE are explainable. Importantly, explainability ensures that models are fair, trustworthy, and valuable to scientific understanding [24]. PHASE takes a step in the direction of allowing scientists to collaborate on EHR data which is typically accessible by only a single group (data silos [25]) by investigating approaches to train embedding models th at generalize to unseen data.

## Results

### Five perioperative outcomes from three hospital datasets

We are interested in forecasting important outcomes associated with surgical morbidity. The first is hypoxemia (i.e., low blood oxygen level), a historically important risk factor associated with anesthesia-related morbidity [26–28], that has been shown to result in harmful effects on nearly every end organ in a variety of animal models [29, 30]. The next three outcomes are hypocapnia (i.e., low blood carbon dioxide), hypotension (i.e., low blood pressure), and hypertension (high blood pressure). Negative physiological effects associated with hypocapnia include reduced cerebral blood flow and reduced cardiac output [31] and intra-operative hypocapnia is associated with delays in the return of spontaneous respiration, increased probability of post-operative nausea and vomiting, and postoperative cognitive dysfunction [32, 33]. Prolonged episodes of perioperative hypotension are associated with end-organ ischemia as well as assorted other adverse postoperative complications [34–37]. In addition, perioperative hypertension has been tied to increased risk of postoperative intracranial hemorrhage in craniotomies [38] and end organ dysfunction [39]. Although it is impossible to design experiments aimed at identifying causality of morbidity or post-operative complications, our outcomes represent important and well-known risk factors. Phenylephrine is a medication frequently used to treat hypotension during anesthesia administration [40]. Epinephrine is often used as an additive in local anesthetics (to improve the depth and duration of the anesthesia), as well as to reduce bleeding [41]. Predicting phenylephrine and epinephrine use lets us further evaluate PHASE because they represent clinical decisions rather than an aspect of patient physiology as in the previous outcomes.

To evaluate our methodology with these outcomes, we utilize data from three different hospital datasets, summarized in Table 1 (Methods section “Datasets” and Supplementary Note 2). In brief, we consider two operating room datasets from distinct medical centers which we denote as OR_0_ and OR_1_. We also use the publicly available intensive care unit MIMIC-III dataset which we refer to as ICU_M_ [20]. As inputs, we use fifteen physiological signal variables: *SAO2* Blood oxygen saturation, *ETCO2* End-tidal carbon dioxide, *NIBP[S/M/D]* Non-invasive blood pressure (systolic, mean, diastolic), *FIO2* Fraction of inspired oxygen, *ETSEV/ETSEVO* End-tidal sevoflurane, *ECGRATE* Heart rate from ECG, *PEAK* Peak ventilator pressure, *PEEP* Positive end-expiratory pressure, *PIP* Peak inspiratory pressure, *RESPRATE* Respiration rate, *TEMP1* Body temperature in addition to six static variables: Height, Weight, ASA Code, ASA Code Emergency, Gender, and Age. All variables are consistently measured in the operating room datasets, but only *SAO2* is consistently measured in the ICU dataset.

**Table 1:** Training set statistics for different data sources.

Dataset	OR_0_	OR_1_	ICU_M_
Department	OR	OR	ICU
Number of procedures/stays	29,035	28,136	1,669
Gender (% female)	57%	38%	44%
Age (yr) Mean	51.859	48.701	63.956
Age (yr) Std.	16.748	18.419	17.708
Weight (lb) Mean	185.273	181.608	176.662
Weight (lb) Std.	54.042	54.194	55.448
Height (in) Mean	66.913	67.502	66.967
Height (in) Std.	8.268	8.607	6.181
ASA Code Emergency	7.65%	15.31%	-
Hypoxemia Base Rate	1.09%	2.19%	3.93%
Hypocapnia Base Rate	9.76%	8.06%	-
Hypotension Base Rate	7.44%	3.53%	-
Hypertension Base Rate	1.70%	1.66%	-
Phenylephrine Base Rate	10.57%	10.95%	-
Epinephrine Base Rate	4.73%	7.71%	-

Each outcome has a different number of samples due to missing data.

Our metric of evaluation is the area under a precision recall curve, otherwise known as average precision (AP), which is more informative than the area under a receiver operating curve (ROC AUC) for binary predictions with low base rates [42], as in the outcomes we consider. In particular, we focus on the percent improvement over using the raw, unprocessed physiological signals as an evaluation metric, which is analogous to transfer loss: the difference between the transfer error and the in-domain baseline error [43]. We additionally report the absolute value of the AP (and ROC AUC for a subset of results) in Supplementary Discussion section “Results in AP and ROC AUC scale”.

### Overview of the PHASE framework

**Fig. 2:**
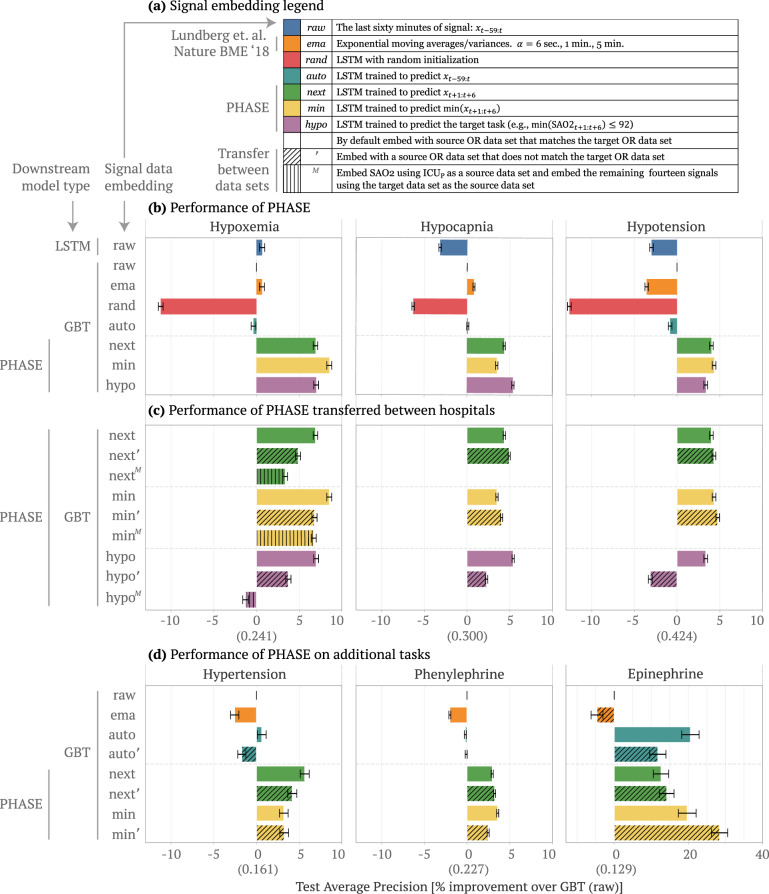
Performance of PHASE embedding models. Comparing the performance of downstream models trained with different embeddings of physiological signals concatenated to static features. We report the average precision (% improvement over GBT model trained with *raw* signal data, 99% confidence intervals from bootstrapping the test set). We use OR_0_ and OR_1_ as target datasets and then aggregate across both by averaging the resultant means and standard errors of the % improvement. **a** The upstream embedding models we use to extract the physiological signal features where raw is the identity function, ema is an exponential moving average, and the rest are LSTMs trained in specific ways.**b** The performance of downstream prediction models for a variety of standard embedding approaches (when the source hospital is the same as the target hospital). We compare combinations of downstream models and embeddings for three adverse surgical outcomes (hypoxemia, hypocapnia, and hypotension). **c** The performance of transferred embedding (*next*', *next*^*M*^, *min*', *min*^*M*^, *hypo*', and *hypo*^*M*^) vs. non-transferred (*next*, *min*, and *hypo*) models for the above three adverse outcomes. In the transferred approaches the source hospital is different to the target hospital. **d** Performance of approaches for standard and transferred embedding on additional outcomes: hypertension (high, rather than low, blood pressure); phenylephrine and epinephrine (doctor action prediction). We do not evaluate *hypo* embeddings in this setting, because the outcomes are not “hypo” events. Model architectures in Supplementary Note 6. We report the average precision value of the *raw* model in parenthesis on the *x*-axis.

**Fig. 3:**
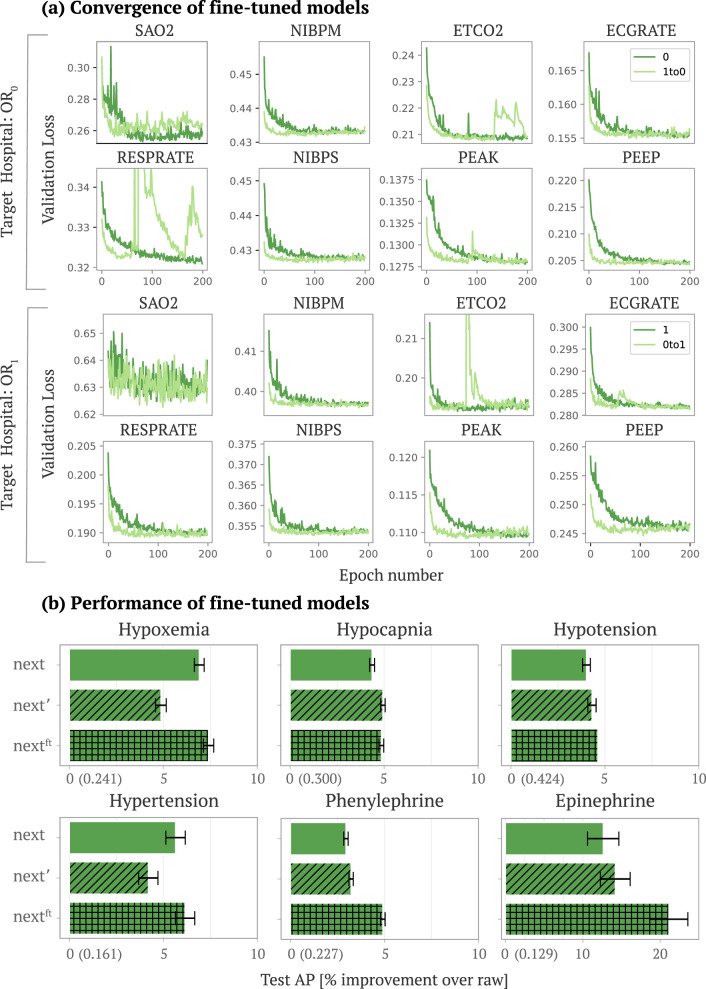
Performance of fine-tuned embedding models. **a** The convergence of fine-tuned models. The top eight plots fix OR_0_ as the target dataset (we plot eight out of the total fifteen signals). Dark green lines show the convergence of a randomly initialized LSTM trained in OR_0_ and light green show the convergence of an LSTM trained in OR_0_ initialized using weights from the best model in OR_1_ (fine-tuning). The bottom two rows show the analogous plots with OR_1_ as the target dataset. Because deep models are typically trained iteratively using some variant of stochastic gradient descent, convergence plots are used to assess the convergence of deep models as a function of the number of iterations (epochs) based on the performance on a held out validation set (validation loss). **b** The performance of GBT models trained on embeddings from standard embedding models (*next*), transferred embedding models (*next*'), and fine-tuned embedding models (*next*^*ft*^) (best models from light green in (**a**)). We report the average precision value of the *raw* model in parenthesis on the *x*-axis.

PHASE is an approach to embed physiological signals. We consider an embedding framework using *upstream embedding models* *U* that are trained for each physiological signal in a source hospital dataset *H*_*s*_. We evaluate upstream embedding models with a downstream prediction model *D* whose inputs are the embedded physiological signals concatenated to static variables and outputs are adverse surgical outcomes. *D* is trained in a target hospital dataset *H*_*t*_. We evaluate our models in three ways (Fig. 1c): (1) standard embedding where the source hospital is the same as the target hospital *H*_*s*_ = *H*_*t*_ (Fig. 2b, d), (2) transferred embedding where the source hospital is different to the target hospital *H*_*s*_ ≠ *H*_*t*_ (Fig. 2c, d), and (3) fine-tuned embedding where the upstream embedding model is first trained to convergence in a different source hospital *H*_*s*_ ≠ *H*_*t*_ and then used to initialize a model that is trained to convergence in the target hospital *H*_*s*_ = *H*_*t*_ (Fig. 3).

The modeling decision of *per-signal* upstream embedding was driven by several advantages: (1) we showed that per-signal embedding models produce embeddings that outperform downstream prediction models trained on the raw signals or hand-engineered signal features (described in Results section “Comparing approaches to embed physiological signals”) (2) we found that per-signal embedding models worked better than a single embedding model trained on all signals jointly in (Supplementary Discussion section “Benchmarking against a jointly trained embedding model”), and (3) we demonstrate that per-signal embedding models work even in a heterogeneous setting where the variables available in the target hospital are different to the variables available in the source hospital (Supplementary Discussion section “Applying PHASE for heterogeneous features”).

Here, we briefly describe the embeddings: *raw, ema, rand, auto, next, min*, and *hypo* in Fig. 2a (more details in Methods section “Set-up”). *Raw* and *ema* are not deep learning models. Instead, *raw* is the raw signal itself and *ema* are exponential moving averages and variance features from Lundberg et al. [21]. The remaining embeddings all use the final hidden layer of LSTMs trained in a source hospital *H*_*s*_ to embed the signals. The first embedding is *rand*, which uses an untrained LSTM with random weights. The second is an unsupervised approach called *auto*, which uses an LSTM trained to autoencode the input. The following two approaches (*next* and *min*) are self-supervised: the LSTM outputs are drawn from the same physiological signal variable as the input, but are taken from different parts of the signal. *Next* uses LSTMs trained to predict the next 5 min of a particular signal; *min* uses LSTMs trained to predict the minimum of the next 5 min of a particular signal. The final approach, *hypo*, is a traditional supervised approach to transfer learning where the embedding model has the same output as the downstream prediction model (either hypoxemia, hypocapnia, or hypotension).

### Comparing approaches to embed physiological signals

As a start, we first compare two popular machine learning models (GBTs and LSTMs) trained on the raw signal data (i.e., without embedding) concatenated to static patient data. In this section we will refer to results according to (1) the downstream model type and (2) the signal embedding type (for instance, GBT *raw* denotes a gradient boosted tree model trained with the raw minute by minute signal data). In Fig. 2b, GBT *raw* performs comparably to LSTM *raw* for hypoxemia and better for hypocapnia and hypotension even though the LSTM should be more suitable to the time series signal data. Based on prior literature, we hypothesize that the GBT better captures patterns in the static patient data which is tabular [23], but the LSTMs better capture patterns in the time series data. In order to leverage the advantages of both model types, we propose PHASE which utilizes LSTMs to embed physiological signals and GBTs to perform the final prediction using the extracted features concatenated to static patient data (Fig. 1a). In the following sections we primarily use GBTs as the downstream model and when we refer to our results solely by the signal data embedding they are assumed to use GBTs as the downstream model (for instance, *next* denotes a GBT model trained with *next* embedded data).

We first evaluate the PHASE methods that include two self-supervised embeddings (*next* and *min*) and a supervised embedding (*hypo*) in a standard embedding setting where the source dataset is the same as the target dataset (Fig. 2b). We train GBT downstream models on the physiological signal embeddings concatenated to static patient features to see if the embeddings are more informative than the raw signals. *Rand* (which serves as a lower bound) transforms physiological signals in an uninformative manner and makes it harder to predict the outcomes of interest in comparison to the raw signals. Furthermore, *ema* and *auto* fail to consistently improve or impair performance relative to *raw* and thus are not viable features. In contrast, the PHASE methods (*next*, *min*, and *hypo*) consistently yield models that outperform the alternative approaches across all three outcomes (all *p*-values < 0.05). In particular, *ema* is a gradient boosted tree model trained with hand engineered features (exponential moving average) shown to be on par with practicing anesthesiologists at forecasting hypoxemia (Lundberg et al. Nature BME 2018 [21]). PHASE embeddings further improve over this approach suggesting that PHASE outperforms clinicians for forecasting hypoxemia by approximately 5% (Fig. 2b).

In order to see how the choice of embedding model output affects downstream model performance we can take a closer look at *auto*, *next*, *min*, and *hypo*. Contrasting PHASE embeddings to *auto* suggests that *incorporating the future in the source task is crucial* (as in *next*, *min*, and *hypo*). However, while taking the minimum (*min*) and thresholding (*hypo*) make the upstream embedding model’s outcome more similar to the downstream prediction model’s outcome, *min* and *hypo* embeddings do not consistently improve downstream prediction performance compared to *next*.

The previously described results show that PHASE works when forecasting hypoxemia, hypocapnia, and hypotension; however these outcomes are all associated with low signals (hence the “hypo” prefix). In order to validate that PHASE performs well for “non-hypo” outcomes as well, we consider three additional outcomes: hypertension (i.e., high blood pressure), phenylephrine administration, and epinephrine administration (doctor action prediction) (Fig. 2d). For hypertension we empirically demonstrate that *next* embeddings are better than *min* embeddings. This is to be expected because *min* focuses on the minimum of the future signal, whereas hypertension is defined as blood pressure being too high and it therefore addresses the maximum of the future signal. For phenylephrine, both the *next* and *min* models improve over standard approaches. One potential reason is that phenylephrine is typically administered in response to low blood pressure and thus *min* models are relevant to phenylephrine administration. For epinephrine, *auto*, *next*, and *min* models all improve over *raw* and *ema*. Interestingly, auto improves over alternative approaches, perhaps due to the low sample size for the epinephrine outcome (Supplementary Table 2). However, *auto* is not the best approach overall, because only *next* and *min* consistently improve over *raw* and *ema* approaches for the other outcomes.

### Evaluating upstream embedding models on unseen data

Previously we focused on a standard embedding setting in a single medical center; in this section, we examine the performance of PHASE when the upstream LSTM embedding models are trained in one dataset but used to embed signals in an unseen dataset (i.e., *transferred* embedding setting). We analyze two distinct transfer learning settings where the source hospital differs to the target hospital (more details in Methods section “Transferred embedding”). We utilize a superscript notation ($^{\prime}$ and ^*M*^) to denote transfer learning. The apostrophe ($^{\prime}$) denotes that we trained LSTMs in one operating room dataset and then fixed them to embed signal variables and evaluate performance with a downstream GBT model in the other. The superscript M (^*M*^) denotes that we trained the LSTM for SAO2 in ICU_M_ and the other LSTMs in the target dataset. Note that MIMIC-III (ICU_M_) has high rates of missingness for signals except for ECG (which is not directly present in the OR datasets) and SAO2. This means we were able to train an upstream LSTM only for SAO2 from ICU_M_ and we extracted features from the remaining signals using LSTMs trained in the target domain. This result is still meaningful, because it means we can use upstream embedding models trained in different domains synergistically.

Training the LSTM embedding models on a source dataset that differs from the target dataset and using a GBT downstream model ($^{\prime}$ and ^*M*^ in Fig. 2c, d) generally outperforms conventional approaches: the LSTM trained on raw data and the GBT trained on raw or engineered features (LSTM *raw*, GBT *raw*, and *ema* in Fig. 2b, d). The *next* and *min* embeddings in the transferred embedding settings (*next*$^{\prime}$, *min*$^{\prime}$, *next*^*M*^, *min*^*M*^) outperform the conventional approaches for all possible outcomes (Fig. 2c) including hypertension, phenylephrine, and epinephrine (Fig. 2d). However, for *hypo*, the supervised embedding, *hypo*$^{\prime}$ improves over *raw* embeddings for hypoxemia and hypocapnia, but actually hurts performance for hypotension. Furthermore the *hypo*^*M*^ embedding also hurts performance for hypoxemia relative to using the *raw* embedding. This suggests that the choice of LSTM embedding model output is important and the supervised learning outcome (*hypo*$^{\prime}$, *hypo*^*M*^) does not generalize to unseen data as well as the self-supervised approaches (*next*$^{\prime}$, *next*^*M*^, *min*$^{\prime}$, *min*^*M*^).

Comparing the transferred embedding models ($^{\prime}$ and ^*M*^ in Fig. 2c, d) to the standard embedding models (*next*, *min*, *hypo* in Fig. 2c, d) we see that the transferred embedding models generally perform comparably to the standard embedding models even though they are evaluated on previously unseen data. In particular, we see that the *next*$^{\prime}$, *min*$^{\prime}$, *next*^*M*^, and *min*^*M*^ embeddings perform comparably to their standard, non-transferred counterparts (*next* and *min*). It is worth noting that the transferred embeddings are equally performant for hypocapnia and hypotension; however, slightly reduce downstream performance for hypoxemia and hypertension, which may be due to differences in the hospital datasets (e.g., covariate shift). As before, we see that the *hypo*$^{\prime}$ and *hypo*^*M*^ embeddings perform substantially worse than their non-transferred counterpart *hypo*.

Although transferred PHASE embeddings perform slightly worse in the hypoxemia and hypertension prediction settings, one important advantage of transferring models is that end users in the target domain can use them at *no additional training cost*. Training all upstream LSTM embedding models for *next* constituted roughly 66 hours on an NVIDIA GeForce RTX 2080 Ti GPU. Clinicians who lack either computational resources or deep learning expertise to train their own models from scratch can instead use an off-the-shelf, fixed embedding model. Given that machine learning is usually not the primary concern of hospital staff, fixed embedding models are a straightforward way to improve the performance of models trained on physiological signal data at minimal cost to the end users.

There are two additional considerations for transfer learning: (1) In our results, we focus on evaluation using GBT downstream models. In order to show that the features we extract consistently boost performance and are robust to the choice of the downstream model we replicate our results for a multilayer perceptron (MLP) downstream model in Supplementary Discussion section “MLP downstream model”. (2) Per-signal LSTM embedding models outperform a single LSTM embedding model jointly trained with all signals in Supplementary Discussion section “Benchmarking against a jointly trained embedding model”. However, per-signal embedding models have an additional advantage: they work even when the variables available in the target hospital do not exactly match the ones in the source hospital (*feature heterogeneity*). Per-signal LSTM embedding models work in heterogeneous settings because end users can pick and choose models that correspond to the signals available at their institution. In comparison, a model trained on all possible variables would be unusable on a new hospital dataset with different variables. In Supplementary Discussion section “Applying PHASE for heterogeneous features”, we show that in heterogeneous settings where the target hospital has fewer features than the source hospital, GBTs trained with PHASE consistently outperform GBTs trained with the raw signals.

### Fine-tuning upstream embedding models improves performance and reduces computational cost

In Results section “Evaluating upstream embedding models on unseen data” we discussed that using PHASE embedding models in the transferred embedding setting are preferable to the standard embedding setting in terms of training cost; however, the standard embedding models still showed slightly better performance for hypoxemia and hypertension. Alternatively, we propose a fine-tuned embedding approach where we assume an end user in the target hospital has been provided a pre-trained embedding model trained in a distinct source hospital. Fine-tuning posits that deep models initialized using pre-trained models from a separate domain work better than randomly initialized models [44]. We train PHASE in a fine-tuning setting where upstream embedding models are trained in an OR target hospital initialized using the weights from the best model from the other OR hospital dataset (detailed setup in Methods section “Fine-tuned embedding”).

We find that PHASE in the fine-tuned embedding setting boosts performance over both standard embedding (Results section “Comparing approaches to embed physiological signals”) and transferred embedding (Results section “Evaluating upstream embedding models on unseen data”) in Fig. 3b. We focus on *next* for the following experiment because it performed and generalized well across most outcomes in previous sections. In Fig. 3, we evaluate the convergence and performance of fine-tuning LSTM embedding models. Figure 3a shows the convergence of fine-tuned models. The top two rows fix OR_0_ as the target dataset. Dark green lines show the convergence of a randomly initialized LSTM and light green show the convergence of an LSTM initialized using weights from the best model in OR_1_. The bottom two rows show the analogous plots with OR_1_ as the target dataset. In Fig. 3a we see that fine-tuning LSTMs rather than training them from scratch consistently leads to much faster convergence. In Fig. 3b, we see that LSTMs obtained from fine-tuning (*next*^ft^) consistently outperform those trained in a single dataset: standard embeddings (*next*) and transferred embeddings (*next*$^{\prime}$). These results indicate that end users can fine-tune PHASE LSTMs to boost performance at lower computational cost in comparison to training models from scratch. Although fine-tuning is more computationally costly than a pre-trained model (transferred embedding), the performance gains from fine-tuning are more consistent.

### Validating models with local feature attributions

**Fig. 4:**
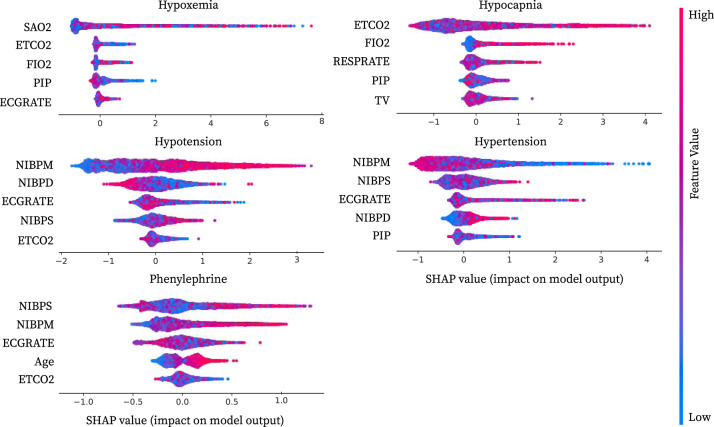
Visualization of important physiological variables. Local feature attribution summary plots for the top five most important variables from GBT models trained with *next* embeddings in the target dataset OR_0_. In order to obtain attributions for each variable we explain each GBT using Interventional Tree Explainer. This gives us attributions for *next* embeddings for the fifteen physiological signal variables (200 dimensional embeddings for each) and six static variables. We sum over embedding attributions to obtain the importance of a particular physiological signal variable. Summing over the attributions guarantees that we maintain the axiom of efficiency (Methods section “Local Feature Attributions”). On the *x*-axis we report this aggregated attribution value that indicates the variable’s cumulative impact on the model output. The colors of the points are either the feature’s value for static variables or the sum over all *next* embeddings for a given physiological signal variable. More detailed attributions in Supplementary Discussion section “Full summary plots”.

We summarize key variables used by downstream GBT models using summary plots (Fig. 4). In these plots, each point represents a feature’s importance for a single sample, with the *x*-axis showing the feature’s impact on the model’s output and the colors indicates the feature’s value (attribution method details in Methods section “Local Feature Attributions”). We focus on explaining GBT models trained on PHASE *next* embeddings in terms of each variable because *next* embeddings were performant across most of the outcomes we considered. The colors are the sum of all features associated with a single signal variable (200 extracted features) which are not naturally interpretable because the embedding values can be arbitrarily positive or negative based on the embedding models.

Standard approaches to train embedding models would use all signal variables as inputs to a single model. These approaches are harder to interpret, because each embedding dimension may be dependent on multiple signals simultaneously. Having per-signal embedding models as in PHASE allows us to clearly interpret each embedding as being dependent on a single physiological signal variable.

We validate important variables against prior literature for models trained on *next* embeddings for all five outcomes (Fig. 4). For hypoxemia, the important variables includes variables logically connected to blood oxygen: *SAO2*, *ETCO2*, and *FIO2* are all associated with the respiratory system, while *PIP* is tied to mechanical ventilation which is naturally linked to blood oxygen [45, 46]. For hypocapnia *ETCO2* is logically the most important feature. Furthermore, using *FIO2*, *RESPRATE*, *PIP*, and TV to forecast hypocapnia makes sense because these variables all relate to either ventilation or respiration. As one would expect, for hypotension and hypertension, key variables are generally the three non-invasive blood pressure measurements: *NIBPM*, *NIBPD*, *NIBPS*. Furthermore, a number of studies validate the importance of *ECGRATE* (heart rate measured from ECG signals) to forecasting hypotension and hypertension [47, 48]. Finally, phenylephrine is typically administered during surgery in response to hypotension, thus validating the importance of *NIBPS*, *NIBPM*, and *ECGRATE*. Similarly, age being more important to forecast phenylephrine use may be tied to its predictive relationship to hypotension as well as anesthesiologists’ heightened vigilance to hypotension in the higher-risk older population [49].

## Discussion

This study explored machine learning techniques for forecasting adverse surgical outcomes. Based on our findings, one possible use case for PHASE embeddings is to improve the accuracy of machine learning derived early warning software systems [50] by alerting attending anesthesiologists. Given the rates of adverse events in the operating room [3–5], computational forecasting that provides advanced warning may be of widespread utility to medical practitioners. This is especially the case given that the outcomes we considered (hypoxemia, hypocapnia, hypotension, and hypertension) are all tied to a number of harmful physiological effects.

This work also shows physiological signal embeddings are effective in several settings. We demonstrate that standard embedding using LSTMs improves the performance of downstream models (GBT and MLP), which implies that pipelines utilizing deep networks to embed physiological signals are effective for electronic healthcare record data. Next, we show that PHASE embedding models work almost equally well in a transferred embedding setting as in a standard embedding setting, and, in fact, work better than randomly initialized models if fine-tuned. This implies that sharing pre-trained networks can improve downstream models in terms of computational needs and predictive performance. Furthermore, we found that embedding models trained on ICU data performed surprisingly well, which aligns with our findings that *next* models performed better than *hypo* models during transference. Both of these findings point to the hypothesis that the majority of improvement from PHASE is due to self-supervision with future signals, rather than necessarily having similar distributions of adverse events (which likely differ between hospital settings).

PHASE uses independently trained LSTMs for each signal variable. Surprisingly, we demonstrate that our per-signal approach outperforms a jointly trained embedding model LSTM (see Supplementary Discussion section “Benchmarking against a jointly trained embedding model”). Furthermore, having each LSTM associated with a single physiological signal actually proves to be an advantage. Hospitals often collect different sets of physiological signal variables; to address this heterogeneity, target hospitals with different but overlapping variables to a source hospital can use the embedding models for the variables which they both have (see Supplementary Discussion section “Applying PHASE for heterogeneous features”). In addition to measuring different physiological signals, different hospitals may encounter substantially different patients. To better investigate our results, we report the average precision stratified by the top ten diagnoses for each target OR dataset and by the ASA physical statuses in Supplementary Discussion section “Evaluating by ASA physical status and diagnosis”. Finally, embedding models are frequently used to improve predictions in smaller target datasets as in [51]. We include an evaluation of PHASE in this setting in Supplementary Discussion section “Evaluating *next* models in a smaller target dataset”.

One limitation of PHASE is that although sharing models reveals less information than sharing data, it is possible to use model inversion attacks on the PHASE embedding models [52] to find physiological signals similar to the training data. Although we attempted to use differentially private versions of stochastic gradient descent [53] to train our embedding models, the randomness inserted in the training process made it difficult to train effective models. We leave investigation and development of effective privacy preserving techniques to train such models to future work. Another limitation of our data is that the embedding models only apply to physiological signals sampled once per minute. We leave exploration of adapting models to accommodate multiple sampling frequencies and irregularly sampled signals to future work as well because they would likely require resampling (decimation/interpolation) or ML models that accommodate irregular patterns of missingness. Additionally, it should be said that there is complementary work discussing deep learning for electrocardiograms [54, 55] and electroencephalograms [56]. We focus primarily on minute by minute physiological signals collected within an operating room setting. As such, although we do have an ECGRATE variable, we do not directly use the electrocardiogram signals. An additional limitation of our experiments is that there are many possible thresholds that can be used to define hypoxemia, hypocapnia, hypotension, and hypertension. While our goal in this manuscript is not to identify the best possible thresholds for each of these outcomes, this is a research direction that would be important prior to any attempt at deploying machine learning systems that forecast these outcomes. To take a step in making sure PHASE is robust to thresholds, we evaluate PHASE against alternative definitions of hypoxemia, hypocapnia, and hypotension in Supplementary Discussion section “Evaluating alternative outcome definitions”. A final potential future direction is to generate per-user embeddings as in Spathis et al. In our experiments, simply aggregating embeddings across the time dimension is likely to lose information important to predicting our time-dependent outcomes. Alternative approaches might include per-user fine-tuning and incorporating user IDs or demographics into the training process of upstream embedding models.

Our work takes an important step forward in applying machine learning to the domain of physiological signals. Previous approaches utilize self-supervised techniques similar to *next* and *auto* in video sequences [57], NLP [58], and cross-signal prediction of HR from accelerometer signals [59]. Other broad categories of approaches involve data augmentations of accelerometer data aimed towards improving generalization [60, 61] and contrastive learning that focuses on similarity of negative and positive pairs of samples [62–65]. We include a comparison to several of these approaches in the Supplementary Discussion section “Evaluating additional self-supervised approaches”.

Drawing on parallels from computer vision (CV) and natural language processing (NLP), both exemplars of transfer learning, physiological signals are well suited to neural network embeddings (i.e., transformations of original inputs into a space better suited to make predictions). In particular, CV and NLP share two notable traits with physiological signals. The first is *consistency*. The CV domain has consistent features: edges, colors, and other visual attributes [66, 67]; the NLP domain uses a particular language with semantic relationships consistent across bodies of text [68]. For sequential signals, we saw that physiological patterns are consistent, because PHASE generalized across hospitals in a transferred embedding setting. The second attribute is *complexity*. Each of these domains is sufficiently complex to make learning embeddings non-trivial. These factors suggest that individual research scientists must make redundant efforts to learn embeddings that may ultimately be very similar. To avoid this problem, NLP and CV have made significant progress on standardizing and evaluating pre-trained models that are often used to generate embeddings [58, 69–72]. Many such pre-trained models are part and parcel of popular deep learning packages (e.g., Keras pre-trained models and PyTorch pre-trained models). In the health domain, similar standardization of physiological signals is natural as well. More significantly, the use of physiological signals is constrained by patient privacy; this makes it difficult to share *data* between hospitals. However, sharing *models* between hospitals does not directly expose patient information. Sharing models in this way could allow machine learning for physiological signals to see similarly large advances as in computer vision and natural language.

## Methods

### Ethics

The data for the OR study data is from institutional electronic medical record and data warehouse systems after receiving approval from the Institutional Review Board (University of Washington Human Subjects Division, Approval no. 46889). Protected health information was excluded from the dataset that was used for the machine-learning methods. We affirm that we have complied with all relevant ethical regulations.

The electronic data for the intensive care unit study data was retrieved from the PhysioNet Clinical Databases after data use agreement approval.

### Datasets

The operating room (OR) datasets were collected via the Anesthesia Information Management System (AIMS), which includes static information as well as real-time measurements of physiological signals sampled minute by minute. OR_0_ was drawn from an academic medical center and OR_1_ from a trauma center. Two marked differences between the patient distributions of OR_0_ and OR_1_ are the gender ratio (57% females in the academic medical center versus 38% in the trauma center) and the proportion of ASA codes that are classified as emergencies (7.65% emergencies versus 15.31%). ICU_M_ is a sub-sampled version drawn from PhysioNet’s publicly available MIMIC dataset, which contains data obtained from an intensive care unit (ICU) in Boston, Massachusetts [20]. Although ICU_M_ data contains several physiological signals sampled at a high frequency, we solely used a minute-by-minute *SAO2* signal for simplicity because many other physiological signals had a substantial amount of missingness (Supplementary Note 4). Furthermore, ICU_M_ contained neonatal data that we filtered out. For all three datasets, any remaining missing values in the signal features were imputed by the mean, and each feature was standardized to have unit mean and variance for training neural networks. We include details about the data acquisition software in Supplementary Note 2. Additional details about the distributions of patients in all three datasets are shown in Table 1 and Supplementary Note 3.

### Set-up

For our datasets, we considered a distribution of hospital stays ${{{\mathcal{P}}}}$. Since we wanted to forecast an adverse event in time, we defined samples by first drawing a hospital stay $P \sim {{{\mathcal{P}}}}$ and then drawing a time point *t* ~ (1, ⋯ , *len*(*P*)). For the rest of this set-up, we assume we are operating with samples *i* defined by *t*, *P*.

### Variables

Many variables are associated with each hospital stay. We distinguished between static variables (that are constant throughout the course of a patient’s stay and are solely determined by *P*) and dynamic variables (that change over time and are determined by *P* and *t*). We partition each sample is (*i* is implicitly determined by *P* and *t*) variables into two distinct sets:

1 $${X}^{i}=\left(\underbrace{{X}_{{s}_{1}}^{i},\cdots ,{X}_{{s}_{6}}^{i}}_{\begin{array}{c}{{\mbox{Static variables}}}\end{array}},\underbrace{{X}_{{d}_{1}}^{i},\cdots ,{X}_{{d}_{15}}^{i}}_{\begin{array}{c}{{\mbox{Dynamic variables}}}\end{array}}\right)$$

The six static variables $({X}_{{s}_{1}}^{i},\cdots \ ,{X}_{{s}_{6}}^{i})$ that do not change over the course of a surgery are: Height, Weight, ASA Code, ASA Code Emergency, Gender, and Age.

Furthermore, we utilized fifteen physiological signals for our dynamic variables (visualized in Supplementary Note 1) (${X}_{{d}_{1}}^{i},\cdots \ ,{X}_{{d}_{15}}^{i}$):

- *SAO2*—Blood oxygen saturation
- *ETCO2*—End-tidal carbon dioxide
- *NIBP[S/M/D]*—Non-invasive blood pressure (systolic, mean, diastolic)
- *FIO2*—Fraction of inspired oxygen
- *ETSEV/ETSEVO*—End-tidal sevoflurane
- *ECGRATE*—Heart rate from ECG
- *PEAK*—Peak ventilator pressure
- *PEEP*—Positive end-expiratory pressure
- *PIP*—Peak inspiratory pressure
- *RESPRATE*—Respiration rate
- *TEMP1*—Body temperature
- *PHENYL*—Whether phenylephrine was administered. We only use this as an output variable and not as an input.
- *EPINE*—Whether epinephrine was administered. We only use this as an output variable and not as an input.

To index the dynamic variables, we used the following notation to denote minutes *a* to *b* (where *b* > *a*) of a particular signal:

2 $${X}_{{d}_{j}}^{i}[a:b]\in {{\mathbb{R}}}^{b-a}$$

### Outcomes

We focused on binary outcomes (i.e., downstream prediction tasks):

3 $${y}^{i}\in \{0,1\}$$

Our adverse events define the outcome as a function (*g*( ⋅ ), *e*. *g*. , *g*( ⋅ ) = *min*( ⋅ ) < *C*) of the next five minutes of a physiological signal (${X}_{{d}_{j}}^{i}$):

4 $${y}^{i}=g({X}_{{d}_{j}}^{i}[t+1:t+5])$$

Specifically, we focused on health forecasting tasks; forecasting tasks facilitate preventive healthcare by helping healthcare providers mitigate risk preemptively [73]. In particular, we considered the following five tasks (which all focus on the next 5 min of surgery):

- *Hypoxemia:* was blood oxygen less than 93?
  5 $$\begin{array}{l}\min (\mathop{X}\nolimits^{i}_{SAO2}[t+1:t+5]) \,<\, 93\end{array}$$
- *Hypocapnia:* was end tidal carbon dioxide less than 35?
  6 $$\begin{array}{l}\min (\mathop{X}\nolimits^{i}_{ETCO2}[t+1:t+5]) \,<\, 35\end{array}$$
- *Hypotension:* was mean blood pressure less than 60?
  7 $$\begin{array}{l}\min (\mathop{X}\nolimits^{i}_{NIBPM}[t+1:t+5]) \,<\, 60\end{array}$$
- *Hypertension:* was mean blood pressure higher than 110?
  8 $$\begin{array}{l}\max (\mathop{X}\nolimits^{i}_{NIBPM}[t+1:t+5]) \,>\, 110\end{array}$$
- *Phenylephrine:* was phenylephrine administered?
  9 $$\begin{array}{r}\max (\mathop{X}\nolimits^{i}_{PHENYL}[t+1:t+5])=1\end{array}$$
- *Epinephrine:* was epinephrine administered?
  10 $$\begin{array}{r}\max (\mathop{X}\nolimits^{i}_{EPINE}[t+1:t+5])=1\end{array}$$

More details about our labeling schemes are in Supplementary Note 5.

### Embeddings (i.e. features)

We define variables (e.g., height, blood oxygen, etc.) separately from embeddings (e.g., height, minute 20 of blood oxygen, etc.) which the downstream prediction models are trained on. Notationally, we denote embeddings as lower case:

$${x}^{i}=({x}_{{s}_{1}}^{i},\cdots \ ,{x}_{{s}_{6}}^{i},{x}_{{d}_{1}}^{i},\cdots \ ,{x}_{{d}_{15}}^{i}).$$

We embed the dynamic variables, with a function ${U}_{{d}_{k};E}$ of the past 60 min of the physiological signal variable:

$${x}_{{d}_{k}}^{i}={U}_{{d}_{k};E}({X}_{{d}_{k}}^{i}[t-59:t]),\forall k\in 1,\cdots \ ,15,E\in \{raw,ema,rand,auto,next,min,hypo\}.$$

We use the static variables as is: ${x}_{{s}_{k}}^{i}={X}_{{s}_{k}}^{i},\forall k\in 1,\cdots \ ,6$. For GBT downstream models we do not transform the static variables; however, for the LSTM downstream models we do normalize them. Unlike dynamic variables, extracting features from the static variables does not significantly improve performance of downstream models.

### Downstream prediction model

The downstream prediction models *D* are used to evaluate different types of embeddings. They are trained on the embedded samples *x*^*i*^ drawn from a target hospital *H*_*t*_. *D* minimizes binary cross entropy loss to forecast adverse outcomes *y*^*i*^ defined as a function of the future 5 min of a physiological signal (for example hypoxemia would be $\min\left(\mathop{X}\nolimits_{{d}_{SAO2}}^{{i}}[t+1:t+5]\right) \,<\, 93$, where ${X}_{{d}_{SAO2}}^{i}[t+1:t+5]$ denotes the future 5 min of the blood oxygen variable for sample *i*).

### Dynamic embedding

For dynamic variables, we made two important decisions. The first was how much of the signal to use. To make fair comparisons, we gave all models access only to the 60 min (see Supplementary Discussion section “Evaluating window size”) of the signal prior to the outcome (which starts at *t* + 1):

11 $${X}_{{d}_{j}}^{i}[t-59:t]$$

The second important decision was how to embed a signal (${X}_{{d}_{j}}^{i}$). Two natural embeddings are: (1) to use the sixty minutes as is (*raw*):

12 $${x}_{{d}_{j}}^{i}={X}_{{d}_{j}}^{i}[t-59:t]\in {{\mathbb{R}}}^{60}$$

- where ${U}_{{d}_{j};raw}$ is the identity function and (2) to use exponential moving averages and variances as the embedding function ${U}_{{d}_{j};ema}$ (*ema*) [21]:
  13 $${x}_{{d}_{j}}^{i}=\left(EMA({X}_{{d}_{j}}^{i}[t-59:t],\alpha =0.1),EMA({X}_{{d}_{j}}^{i}[t-59:t],\alpha =1)\right.,$$
  14 $$\left.EMA({X}_{{d}_{j}}^{i}[t-59:t],\alpha =5),EMV({X}_{{d}_{j}}^{i}[t-59:t],\alpha =5)\right)\in {{\mathbb{R}}}^{4}$$
- where the exponential moving average is defined as:
  15 $$EM{A}_{\tau }=\alpha \times {X}_{{d}_{j}}^{i}[\tau ]+(1-\alpha )\times EM{A}_{\tau -1},\forall \tau \,>\, t-59$$
  16 $$EM{A}_{t-59}={X}_{{d}_{j}}^{i}[t-59]$$
  17 $$EMA({X}_{{d}_{j}}^{i}[t-59:t],\alpha )=EM{A}_{t}$$
- and the exponential moving variance is defined as:
  18 $${\delta }_{\tau }={X}_{{d}_{j}}^{i}[\tau ]-EM{A}_{\tau -1}$$
  19 $$EM{A}_{\tau }=EM{A}_{\tau -1}+\alpha \times {\delta }_{\tau }$$
  20 $$EM{V}_{\tau }=(1-\alpha )\times (EM{V}_{\tau -1}+\alpha \times {\delta }_{\tau }^{2})$$
  21 $$EMV({X}_{{d}_{j}}^{i}[t-59:t],\alpha =5)=EM{V}_{t}$$

### LSTM embedding

To better extract features from (embed) each physiological signal variable (${X}_{{d}_{j}}^{i}$), we utilized per-signal neural networks (LSTMs) trained in a source hospital *H*_*s*_. We utilized an embedding dimension of 200 nodes (Supplementary Discussion section “Evaluating different embedding sizes”) and the embedding from the final time step (Supplementary Discussion section “Evaluating embedding time slices”). The LSTMs ${L}_{{d}_{j};E}^{{H}_{s}}$ are trained for each physiological signal (we show that per-signal embedding models worked better than a single LSTM trained on all signals jointly in Supplementary Discussion section “Benchmarking against a jointly trained embedding model”) to minimize a loss function (dependent on the embedding type *E*) with the past 60 min of signal *d*_*k*_ as the input:

$${{{{\mathcal{L}}}}}_{E}({L}_{{d}_{k};E}^{{H}_{s}}({X}_{{d}_{k}}^{i}[t-59:t]),{y}_{E}^{i})$$

Table 2 describes the different tasks we used to train LSTMs upstream embedding models including the three self-supervised labels (*next*, *min*, *hypo*) we proposed in PHASE. More specifically, ${U}_{{d}_{j};E}=h\circ {L}_{{d}_{j};E}^{{H}_{s}}$, where the composition *h*∘*L* signifies removing the output layer of *L* to obtain a function that maps the past 60 min of *d*_*k*_ to the activations of the final hidden layer in *L*. For the *rand* embedding the models ${L}_{{d}_{k};rand}$ are LSTM models with random weights. There is no source hospital, because the models are not trained. Then, *auto*, *next*, and *min* embeddings set ${{{{\mathcal{L}}}}}_{E}$ to mean squared error. However, the outcomes differ for each: ${y}_{auto}^{i}={X}_{{d}_{k}}^{i}[t-59:t],{y}_{next}^{i}={X}_{{d}_{k}}^{i}[t+1:t+5],{y}_{mind}^{i}=\min (\mathop{X}\nolimits_{{d}_{k}}^{{i}}[t+1:t+5])$ (note that these outcomes are self-supervised). Finally, *hypo* embeddings set ${{{{\mathcal{L}}}}}_{E}$ to binary cross entropy loss and the outcome is set to be the same as the downstream task *y*^*i*^. Since several of our downstream outcomes were tied to too-low (“hypo”) signals, the approaches in Table 2 were ordered by distance to the downstream task.

**Table 2:** Inputs and outputs for our per-signal upstream LSTMs.

*E*	Domain	Range (Upstream Task)	${{{{\mathcal{L}}}}}_{E}$
*rand*	${X}_{{d}_{j}}^{i}[t-59:t]\in {{\mathbb{R}}}^{60}$	${{\emptyset}}$	${{\emptyset}}$
*auto*	${X}_{{d}_{j}}^{i}[t-59:t]\in {{\mathbb{R}}}^{60}$	${X}_{{d}_{j}}^{i}[t-59:t]\in {{\mathbb{R}}}^{60}$	MSE
*next*	${X}_{{d}_{j}}^{i}[t-59:t]\in {{\mathbb{R}}}^{60}$	${X}_{{d}_{j}}^{i}[t+1:t+5]\in {{\mathbb{R}}}^{5}$	MSE
*min*	${X}_{{d}_{j}}^{i}[t-59:t]\in {{\mathbb{R}}}^{60}$	$\min(\mathop{X}\nolimits_{{d}_{j}}^{{i}}[t+1:t+5])\in {{\mathbb{R}}}^{1}$	MSE
*hypo*	${X}_{{d}_{j}}^{i}[t-59:t]\in {{\mathbb{R}}}^{60}$	*y*^*i*^ ∈ {0, 1}	BCE

We denote embedding names in italics.

We used the following notation to denote an LSTM trained to convergence using ${X}_{{d}_{j}}^{i}$ drawn from the source hospital dataset *H*_*s*_ using inputs and outputs specified by the task in Table 2:

22 $${L}_{{d}_{j};{task}}^{{H}_{s}}$$

As an example, ${L}_{{d}_{j};next}^{{{{\mbox{OR}}}}_{0}}$ indicates that the LSTM was trained for signal ${X}_{{d}_{j}}^{i}$ with inputs ${X}_{{d}_{j}}^{i}[t-59:t]$ and outputs ${X}_{{d}_{j}}^{i}[t+1:t+5]$ on data drawn from OR_0_.

To describe the features associated with the neural network embedding approaches, we removed the output layer of the network and embedded each signal using the final hidden layer of the network. We denote this as:

23 $${x}_{{d}_{j}}^{i}\equiv h\circ {L}_{{d}_{j};next}^{{H}_{s}}({X}_{{d}_{j}}^{i}[t-59:t])\in {{\mathbb{R}}}^{200}$$

where *h* removes the output layer of network *L* and 200 is the number of hidden nodes in *L*.

As an example, if our target dataset was OR_0_, then our physiological signal features for *next* would be:

24 $${x}_{{d}_{j}}^{i}\equiv h\circ \mathop{L}\nolimits_{{d}_{j};next}^{{{{\mbox{OR}}}}_{0}}\left({X}_{{d}_{j}}^{i}[t-59:t]\right)\in {{\mathbb{R}}}^{200}$$

### Transferred embedding

To evaluate transfer learning, we denoted a target hospital dataset *H*_*t*_ (the domain in which we trained the downstream prediction model on embedded variables) and a source hospital dataset *H*_*s*_ (the domain in which we trained our upstream embedding models). In the transference experiments (denoted used superscripts next to the embedding type *E*: *task*$^{\prime}$ and *task*^*M*^) we train our upstream embedding models in a source hospital that is different to the target hospital (*H*_*s*_ ≠ *H*_*t*_).

By default, without the superscript, the source domain matched the target domain (*H*_*s*_ = *H*_*t*_). With an apostrophe, the source domain was the remaining operating room dataset (*H*_*s*_ = OR_0_ if *H*_*t*_ = OR_1_ or *H*_*s*_ = OR_1_ if *H*_*t*_ = OR_0_). As an example, if our target dataset was OR_0_, then our physiological signal features for *next*$^{\prime}$ would be:

25 $${x}_{{d}_{j}}^{i}\equiv h\circ \mathop{L}\nolimits_{{d}_{j};next}^{{{{\mbox{OR}}}}_{1}}\left({X}_{{d}_{j}}^{i}[t-59:t]\right)\in {{\mathbb{R}}}^{200}$$

Finally, for *task*^*M*^, the source domain for the LSTM embedding model for SAO2 was ICU_M_ (*H*_*s*_ = ICU_M_), and the remaining models were trained in a source domain that matched the target domain (*H*_*s*_ = *H*_*t*_). As an example, if our target dataset was OR_0_, then our physiological signal features for *next*$^{\prime}$ would be:

26 $${x}_{{d}_{j}}^{i}\equiv h\circ {L}_{{d}_{j};next}^{{{{\mbox{ICU}}}}_{{{\mbox{M}}}}}({X}_{{d}_{j}}^{i}[t-59:t])\in {{\mathbb{R}}}^{200}\,{{\mbox{for SAO2}}}\,$$

27 $${x}_{{d}_{j}}^{i}\equiv h\circ {L}_{{d}_{j};next}^{{{{\mbox{OR}}}}_{0}}({X}_{{d}_{j}}^{i}[t-59:t])\in {{\mathbb{R}}}^{200}\,{{\mbox{for all other signals}}}\,$$

### Fine-tuned embedding

The fine-tuning approach (denoted as next^ft^) considers fine-tuning models between operating room datasets. If we assume a fixed target dataset *H*_*t*_ = OR_0_. Then, as before, we denote an LSTM trained to convergence on data from OR_1_ to be:

28 $${L}_{{d}_{j};next}^{{{{\mbox{OR}}}}_{1}}$$

For fine-tuning, we used the LSTM trained on samples drawn from OR_1_ (which crucially was not the same as the target dataset) to initialize an LSTM which we then trained until convergence on samples drawn from OR_0_. Notationally, we describe this as:

29 $${L}_{{d}_{j};next}^{{{{\mbox{OR}}}}_{1}\to {{{\mbox{OR}}}}_{0}}$$

The features for dynamic variables under the fine-tuning approach for *H*_*t*_ = OR_0_ were:

30 $${x}_{{d}_{j}}^{i}\equiv h\circ {L}_{{d}_{j};next}^{{{{\mbox{OR}}}}_{1}\to {{{\mbox{OR}}}}_{0}}({X}_{{d}_{j}}^{i}[t-59:t])\in {{\mathbb{R}}}^{200}$$

### Jointly Trained Upstream Model

The jointly trained upstream model (denoted as *next*_m_) involved training an LSTM for several signals simultaneously. To do so, we optimized an LSTM for forecasting the next 5 minutes of all our physiological signals, which we denote as:

31 $${L}_{{d}_{1},\cdots \ ,{d}_{15};next}^{{H}_{s}}$$

Then, the features for dynamic variables under the jointly trained multi-signal model were:

32 $${x}_{{d}_{1}}^{i},\cdots \ ,{x}_{{d}_{15}}^{i}=h\circ {L}_{{d}_{1},\cdots \ ,{d}_{15};next}^{{H}_{s}}({X}_{{d}_{1}}^{i}[t-59:t],\cdots \ ,{X}_{{d}_{15}}^{i}[t-59:t])$$

### Local Feature Attributions

To obtain explanations, we utilized Interventional Tree Explainer, which provides exact Shapley values with an interventional conditional expectation set function (feature attributions with game-theoretic properties) for complex tree-based models [23, 74]. The Shapley values serve as local feature attributions *ϕ*(*f*, *x*^*i*^) that indicate how much each feature in *x*^*i*^ contributed to a single downstream prediction *D*(*x*^*i*^). Positive attribution means that the feature generally increases the output of the model (risk of adverse events) and negative attribution means that the feature generally decreases the output. Shapley values have been used to explain models in a wide variety of applications including biology [75], medicine [76], finance [77], and more.

We sum over local feature attributions to maintain efficiency, one of the desirable axioms Shapley values satisfy [74]. Efficiency loosely states that the attributions for a particular sample sum up to the difference between the model’s prediction and the average model output over the baselines. Efficiency is desirable because it implies that local feature attributions are roughly on the same scale as the model’s output (log-odds, probability-space, etc.). If we average over the attributions for a particular signal, the attributions will no longer satisfy efficiency and attributions for signals will be on a different scale to the attributions for the non-averaged static attributions (height, weight, etc.). In order to guarantee efficiency, we instead sum over the attributions for dynamic (physiological signal features) in order to keep them comparable to the attributions for the static features.

### Reporting Summary

Further information on research design is available in the Nature Research Reporting Summary linked to this article.

## Supplementary information

Supplementary Information

Reporting Summary

## Data Availability

Trained models and performance results from this study are available from the corresponding author upon reasonable request. The ICU dataset is publicly available upon a reasonable request: https://mimic.physionet.org/[20]. The OR datasets from the University of Washington Medical Center and Harborview Medical Center used in the current study are not publicly available, due to institutional restrictions on data sharing and privacy concerns. The de-identified data may be made available to qualified researchers upon reasonable request subject to permission and approval from the corresponding organizations and institutional review boards.

## Code availability

The code for the experiments is available here: https://github.com/suinleelab/PHASE.
