# Supplementary material for: Forecasting adverse surgical events using self-supervised transfer learning for physiological signals
Source: NPJ Digit Med. 2021 Dec 8;4:167. doi: 10.1038/s41746-021-00536-y (PMC8654960; doi:10.1038/s41746-021-00536-y)
Supplement: Supplementary file 1 — Supplementary Information [file 41746_2021_536_MOESM1_ESM.pdf]

1 Supplementary Note 1: Sample time series data

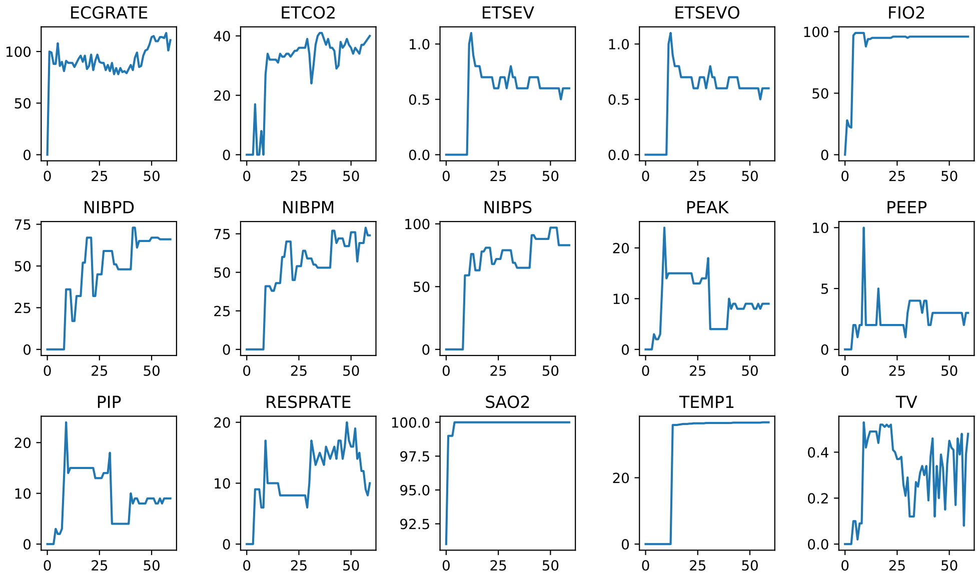

Supplementary Figure 1: *An example of the physiological measurement data.* The x-axis is time and the y-axis differs for each physiological measurement. The physiological signals are derived values rather than the raw waveforms (e.g., SAO2 rather that PPG).

In Supplementary Figure 1, we include an example of the raw signals we use as inputs to our machine learning models. Each physiological signal was measured once a minute, by the data acquisition software described in Supplementary Section 2.

2 Supplementary Note 2: Data acquisition

The OR hospital systems utilize AIMS (Merge AIM, Merge Inc.), a system that automatically captures minute-by-minute hemodynamic and ventilation parameters from the patient monitor and the anaesthesia machine. The system integrates with other hospital EHR systems to automatically acquire laboratory and patient registration information. The automatic data collection is supplemented by manual documentation of medications and anaesthesia interventions to form the complete anaesthesia record during a surgery. For this project, we extracted the high-fidelity anaesthesia data from the AIMS database from May 2012 to June 2014. The medical history data of each patient were also extracted from the hospital systems' EHR data warehouse (Caradigm). The anaesthesia record data and the corresponding medical history data constitute the data we used to evaluate PHASE.

The ICU data is from the MIMIC-III database populated by data acquired during routine hospital care from sources including archives from critical care information systems, hospital EHR database, and the Social Security Administration Death Master file. They utilized two critical care information systems: Philips CareVue Clinical Information System (models M2331A and M1215A; Philips Health-care, Andover, MA) and iMDsoft MetaVision ICU (iMDsoft, Needham, MA) which measure time-stamped nurse-verified physiological measurements and more.

3 Supplementary Note 3: Data distributions

We include figures describing the distributions of weight, height, age (Supplementary Figure 2), and diagnoses (Supplementary Figure 3) for all three datasets.

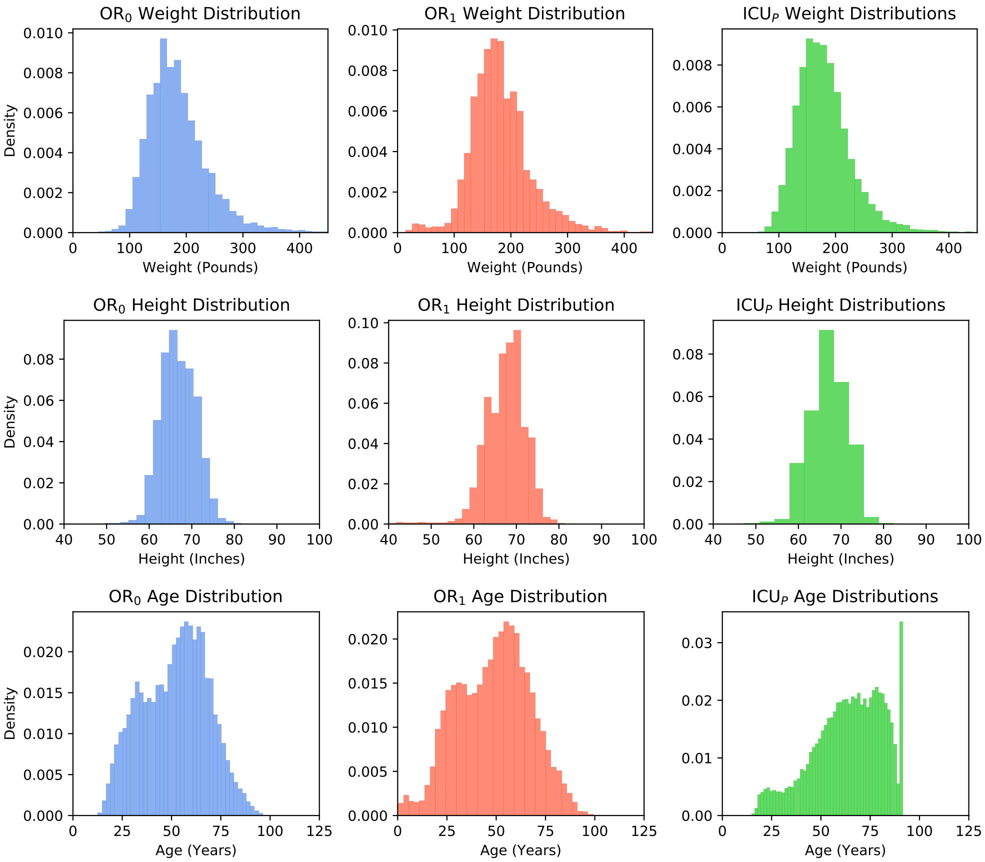

Supplementary Figure 2: *Weight, height, and age distributions.*

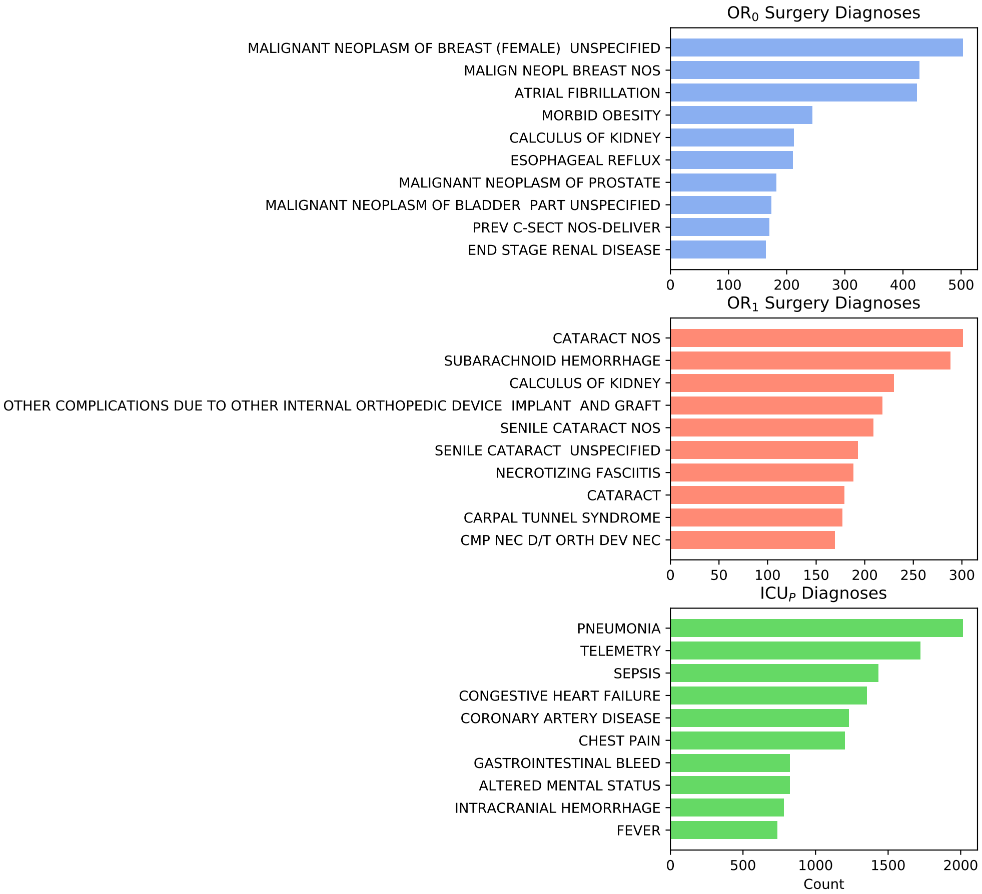

Supplementary Figure 3: *Diagnosis distributions.*

We include figures describing the distributions of ASA physical status, blood loss, procedure duration, and medications for the OR datasets (Supplementary Figure 4).

4 Supplementary Note 4: Availability of ICU_M physiological measurements

In Figure 2, we only utilized SAO2 (SpO2) from the ICU_M data because it was one of the most predictive signals for our outcomes (out of SAO2, ETCO2, and NIBPM) with >80% of available measurements by time points (Supplementary Table 1). By contrast, most other signals had much lower rates of availability and including them would require handling a large amount of missingness by filtering or imputation. To simplify our analysis we focus on generating embeddings only for SAO2 which is highly predictive of the hypoxemia outcome.

Physiological Measurement	Percent of Available Time Points
HR	97.36%
Rhythm Status	93.40%
PVC Rate per Minute	93.31%
RESP	92.82%
Ectopic Status	90.97%
PULSE	86.26%
SpO2	83.95%
NBP Dias	55.37%
NBP Mean	55.37%
NBP Sys	55.37%
ST V	53.41%
ST III	51.03%
ABP Dias	35.14%
ABP Mean	35.14%
ABP Sys	35.14%
CVP	19.12%

Supplementary Table 1: *Minute by minute measurements sorted by availability in* ICU_M.

5 Supplementary Note 5: Labelling

For *hypoxemia*, a particular time point t is labelled to be one if the minimum of the next five minutes is hypoxemic ($\min(SAO2^{t+1:t+6}) \leq 93$). All points where the current time step is currently hypoxemic are ignored ($SAO2^t < 93$). Additionally we ignore time points where the past ten minutes were all missing or the future five minutes were all missing. *hypocapnia*, *hypotension*, and *hypertension* have slightly stricter label conditions. We label the current time point t to be one if ($\min(S^{t-10:t}) > T$) and the minimum of the next five minutes is "hypo" ($\min(S^{t+1:t+5}) \leq T$) (maximum and $\geq$ for hypertension). We label the current time point t to be zero if ($\min(S^{t-10:t}) > T$) and the minimum of the next ten minutes is not "hypo" ($\min(S^{t+1:t+10}) > T$). For *Hypertension*, we use $\max(S^{t+1:t+5}) \geq T$ rather than min and an analogous filtering procedure. All other time points were not considered. For *hypocapnia*, the threshold $T = 34$ and the signal S is *ETCO2*. For *hypotension* the threshold $T = 59$ and the signal S is *NIBPM*. For *hypertension* the threshold $T = 110$ and the signal S is *NIBPM*. Additionally we ignore time points where the past ten minutes were all missing or the future five minutes were all missing. As a result, we have different sample sizes for different prediction tasks (reported in Table **??**). For *phenylephrine* and *epinephrine*, we filter out procedures where they were not administered.

For hypoxemia, we considered SAO2$\leq$ 92% (<93). This threshold coincides with the threshold chosen by a previous machine learning method for forecasting hypoxemia in Lundberg et al. [12]. Furthermore, the specific threshold of 92% falls at the midpoint between the intervention level (< 94%) and emergency

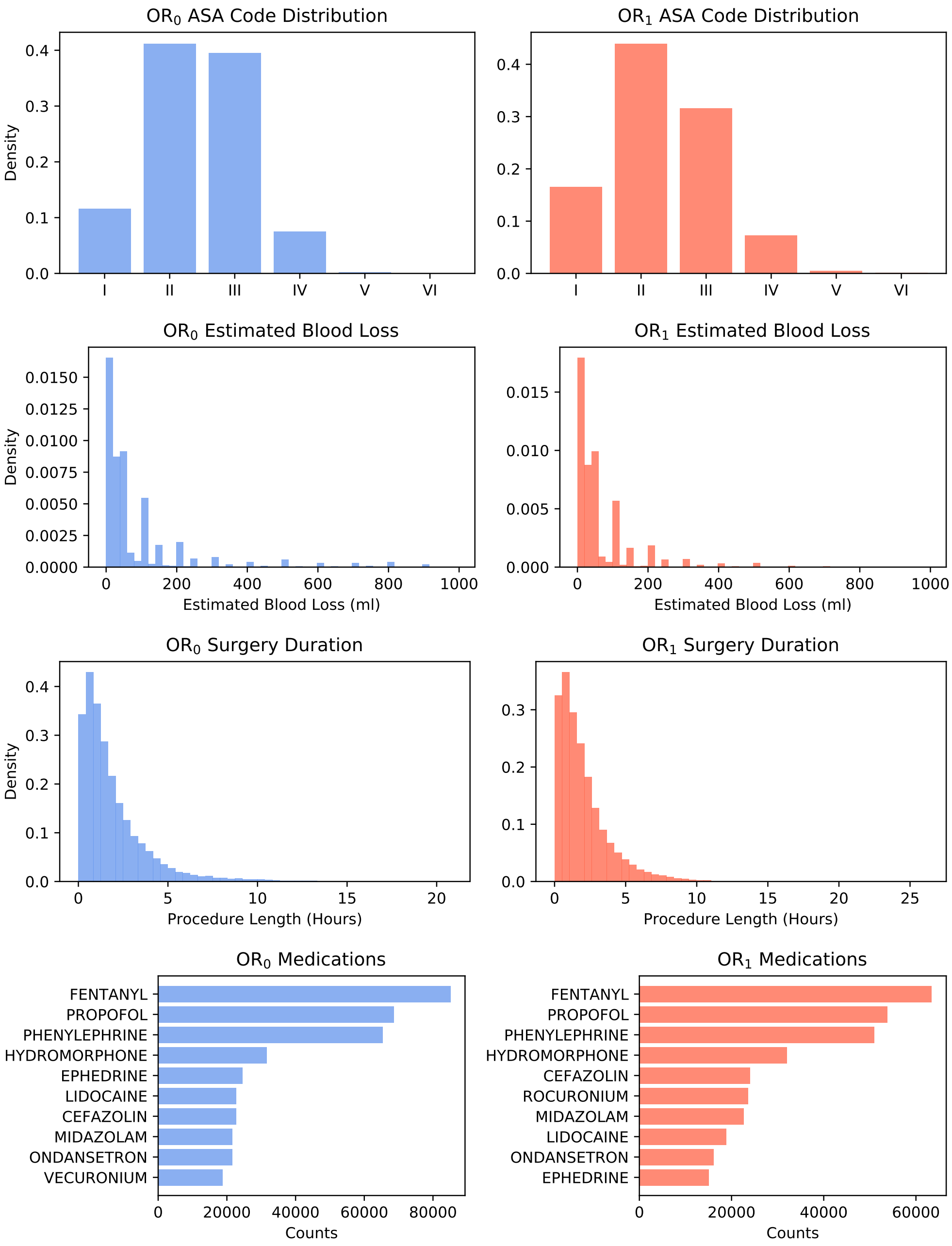

Supplementary Figure 4: *ASA physical status, blood loss, procedure duration, and medications distributions.*

Outcome	n (Hospital 0)	n (Hospital 1)
Hypoxemia	3920564	4167959
Hypocapnia	1259768	1754091
Hypotension	1837676	2332902
Hypertension	2470632	2534954
Phenylephrine	2690484	2004857
Epinephrine	97627	30916

Supplementary Table 2: Sample sizes (train and validation sets) for each outcome. Epinephrine has a much lower sample size because it is rarely administered in comparison to Phenylephrine.

level (< 90%) recommended by the World Health Organization [15] and coincides with Majumdar et al. [13], who identifies associations between SpO2 lower than 92% with major adverse events in pneumonia patients. For hypocapnia, we considered ETCO2<35 mmHg which was found to be associated with increased 30-day mortality rates for five thousand patients ASA I-IV undergoing various surgical procedures (excluding pediatric and cardiac) under general anesthesia [7]. Furthermore, Way and Hill [23] questions the current practice of maintaining mild hypocapnia, instead advocating for mild hypercapnia (ETCO2 around 40 mm Hg or higher). Moreover, although mild hypocapnia is relatively common, an important distinction is that we filter out cases that are currently hypocapnic and focus on forecasting new hypocapnia cases (this is true for all of our signal-derived outcomes). This means that, even though mild hypocapnia is fairly common, we only anticipate new mild hypocapnic cases, resulting in a lower base rate around 8-10%. For hypotension, we considered NIBPM<60 mm Hg which is generally considered clinically significant [17] and approximates the lower bound for proper cerebral autoregulation [2] and myocardial and kidney perfusion [22].

6 Supplementary Note 6: Architecture

6.1 LSTM (upstream embedding) architecture and training

We utilize LSTMs with forget gates, introduced by Gers, Schmidhuber, and Cummins [8], implemented in the Keras library [5] with a Tensorflow back-end [14]. We train our networks with either regression (*auto* and *min* embeddings) or classification (*hypo*) objectives. For regression, we optimize using Adam with an MSE loss function. For classification we optimize using RMSProp with a binary cross-entropy loss function (additionally, we upsample to maintain balanced batches during training). Our model architectures consist of two hidden layers, each with 200 LSTM cells with dense connections between all layers. We found that important steps in training LSTM networks for our data are to impute missing values by the training mean, standardize data, and to randomize sample ordering prior to training. To prevent overfitting, we utilized dropouts between layers as well as recurrent dropouts for the LSTM nodes. We utilized a learning rate of 0.001. Hyperparameter optimization was done by manual coordinate descent. The LSTM models were each run for 200 epochs and the final model was selected according to validation loss. In order to train these models, we utilize three GPUs (GeForce GTX 1080 Ti graphics cards).

6.2 GBM (downstream prediction) architecture and training

We train GBM trees in Python using XGB [3], an open source library for gradient boosting trees. XGB works well in practice in part due to it's ease of use and flexibility. Imputing and standardizing are unnecessary because GBM trees are based on splits in the training data, so that scale does not matter and missing data is informative as is. We train the GBM trees with embedding features from 15 physiological signals, resulting in a total of 3000 features for PHASE methods. In addition, we concatenate static features to the signal features to train and evaluate the models. We found that a learning rate of 0.02 for hypoxemia (0.1 for hypotension and hypocapnia), a max tree depth of 6, subsampling rate of 0.5, and a logistic objective gave us good performance. We fix hyperparameter settings across experiments so that we can focus on comparing different representations our signal data. All XGB models were run until their validation accuracy was non-improving for five rounds of adding estimators (trees). In order to train these models, we utilize 72 CPUs (Intel(R) Xeon(R) CPU E5-2699 v3 @ 2.30GHz)

7 Supplementary Discussion

7.1 Results in AP and ROCAUC scale

In this section, we report the AP scores for each of our main text plots in Supplementary Tables 3-6. We include ±99% confidence intervals. In the main text we report the transfer AP. We additionally report that AP values for a jointly trained model (Supplementary Table 7) and for the heterogeneous feature experiment (Supplementary Table 8. We report a shorter version of the performance/transference results for hypo outcomes in ROC AUC in Supplementary Table 9. We report the performance of additional self-supervised approaches in AP in Supplementary Table 10, the performance of alternative definitions of our hypo outcomes

in AP in Supplementary Table 11, and the performance of *next* variants for a smaller (10%) target data set in AP in Supplementary Table 12.

Downstream Model	Data Type	Hypoxemia	Hypocapnia	Hypotension
LSTM	raw	0.2431 ± 0.0014	0.4109 ± 0.0011	0.2898 ± 0.0009
	raw	0.2411 ± 0.0013	0.4243 ± 0.0012	0.2996 ± 0.0010
	ema	0.2420 ± 0.0013	0.4277 ± 0.0011	0.2890 ± 0.0009
	rand	0.2135 ± 0.0012	0.3973 ± 0.0011	0.2630 ± 0.0009
XGB	auto	0.2402 ± 0.0014	0.4246 ± 0.0011	0.2974 ± 0.0010
	next	0.2585 ± 0.0014	0.4426 ± 0.0011	0.3116 ± 0.0010
	min	0.2621 ± 0.0014	0.4392 ± 0.0011	0.3127 ± 0.0010
	hypo	0.2581 ± 0.0014	0.4471 ± 0.0012	0.3100 ± 0.0010

Supplementary Table 3: Non-transference results for hypo outcomes in AP, corresponding to Figure 2b.

Data Type	Hypoxemia	Hypocapnia	Hypotension
next	0.2585 ± 0.0014	0.4426 ± 0.0011	0.3116 ± 0.0010
next$'$	0.2529 ± 0.0013	0.4451 ± 0.0011	0.3123 ± 0.0010
next^P	0.2486 ± 0.0013		
min	0.2621 ± 0.0014	0.4392 ± 0.0011	0.3127 ± 0.0010
min$'$	0.2580 ± 0.0014	0.4414 ± 0.0012	0.3140 ± 0.0010
min^P	0.2568 ± 0.0014		
hypo	0.2581 ± 0.0014	0.4471 ± 0.0012	0.3100 ± 0.0010
hypo$'$	0.2499 ± 0.0013	0.4342 ± 0.0012	0.2879 ± 0.0010
hypo^P	0.2383 ± 0.0014		

Supplementary Table 4: Transference results for hypo outcomes in AP, corresponding to Figure 2c.

Data Type	Hypertension	Phenylephrine	Epinephrine
raw	0.2275 ± 0.0005	0.1613 ± 0.0013	0.1287 ± 0.0032
ema	0.2227 ± 0.0005	0.1569 ± 0.0012	0.1199 ± 0.0031
auto	0.2270 ± 0.0005	0.1617 ± 0.0012	0.1573 ± 0.0042
auto$'$	0.2271 ± 0.0006	0.1584 ± 0.0012	0.1466 ± 0.0032
next	0.2340 ± 0.0006	0.1703 ± 0.0013	0.1472 ± 0.0035
next$'$	0.2349 ± 0.0006	0.1674 ± 0.0012	0.1482 ± 0.0039
min	0.2355 ± 0.0006	0.1665 ± 0.0012	0.1572 ± 0.0037
min$'$	0.2329 ± 0.0005	0.1662 ± 0.0012	0.1649 ± 0.0040

Supplementary Table 5: Non-hypo outcome results in AP, corresponding to Figure 2d.

7.1.1 Benchmarking against a jointly trained embedding model

In this section, our aim is to investigate whether jointly training the embedding models affords any benefits over training per-signal networks (more details in Section). To do so, we compare the per-signal LSTM embedding models to a much larger LSTM model that is jointly trained. In order to ensure a fair comparison, we used exactly the same hyperparameters (batch size, learning rate, optimizer, number of epochs, etc.) and used roughly the same number of parameters in both cases. For the per-signal models we used 2 layers of 200 LSTM nodes to predict the next five minutes of a given signal (*next*). In the larger LSTM model, we take 15 of these 2 layer networks and concatenate their outputs which is used to predict 15 distinct tasks: the next five minutes of each signal (minimizing mean squared error for all tasks simultaneously). Then we use this jointly trained model to create embeddings for each signal ($next_{\mathrm{m}}$).

Outcome	next	next'	next^{ft}
Hypoxemia	0.2585 ± 0.0014	0.2529 ± 0.0013	0.2597 ± 0.0014
Hypocapnia	0.4426 ± 0.0011	0.4451 ± 0.0011	0.4447 ± 0.0012
Hypotension	0.3116 ± 0.0010	0.3123 ± 0.0010	0.3143 ± 0.0010
Hypertension	0.1703 ± 0.0013	0.1674 ± 0.0012	0.1715 ± 0.0013
Phenylephrine	0.2340 ± 0.0006	0.2349 ± 0.0006	0.2386 ± 0.0006
Epinephrine	0.1263 ± 0.0206	0.1418 ± 0.0197	0.2112 ± 0.0251

Supplementary Table 6: Fine tuning results in AP, corresponding to Figure 3.

Data Type	Hypoxemia	Hypocapnia	Hypotension
next	0.2585 ± 0.0014	0.4426 ± 0.0011	0.3116 ± 0.0010
next'	0.2529 ± 0.0013	0.4451 ± 0.0011	0.3123 ± 0.0010
next	0.2523 ± 0.0014	0.4315 ± 0.0011	0.2829 ± 0.0009
next'_m	0.2486 ± 0.0014	0.4234 ± 0.0011	0.2879 ± 0.0010

Supplementary Table 7: Jointly trained model results in AP, corresponding to Supplementary Figure 5.

Outcome	Number of Signals	raw	next	next'
Hypoxemia	1	0.2413 ± 0.0014	0.2467 ± 0.0014	0.2393 ± 0.0014
	3	0.2412 ± 0.0014	0.2535 ± 0.0014	0.2469 ± 0.0014
	5	0.2402 ± 0.0013	0.2559 ± 0.0014	0.2505 ± 0.0013
	7	0.2417 ± 0.0013	0.2561 ± 0.0014	0.2522 ± 0.0014
	9	0.2414 ± 0.0013	0.2572 ± 0.0014	0.2511 ± 0.0013
	11	0.2393 ± 0.0013	0.2568 ± 0.0014	0.2518 ± 0.0013
	13	0.2414 ± 0.0013	0.2556 ± 0.0014	0.2492 ± 0.0013
	15	0.2419 ± 0.0013	0.2565 ± 0.0014	0.2508 ± 0.0013
Hypocapnia	1	0.3950 ± 0.0011	0.4006 ± 0.0011	0.4009 ± 0.0012
	3	0.4109 ± 0.0012	0.4151 ± 0.0012	0.4149 ± 0.0012
	5	0.4256 ± 0.0012	0.4307 ± 0.0012	0.4312 ± 0.0012
	7	0.4275 ± 0.0012	0.4251 ± 0.0011	0.4319 ± 0.0012
	9	0.4273 ± 0.0012	0.4303 ± 0.0012	0.4352 ± 0.0012
	11	0.4272 ± 0.0012	0.4314 ± 0.0011	0.4390 ± 0.0011
	13	0.4251 ± 0.0012	0.4337 ± 0.0011	0.4386 ± 0.0012
	15	0.4224 ± 0.0012	0.4346 ± 0.0011	0.4410 ± 0.0011
Hypotension	1	0.2590 ± 0.0009	0.2576 ± 0.0009	0.2552 ± 0.0009
	3	0.2722 ± 0.0010	0.2771 ± 0.0009	0.2741 ± 0.0009
	5	0.2828 ± 0.0010	0.3005 ± 0.0010	0.2960 ± 0.0010
	7	0.2948 ± 0.0010	0.3123 ± 0.0010	0.3091 ± 0.0010
	9	0.2952 ± 0.0010	0.3088 ± 0.0009	0.3077 ± 0.0009
	11	0.2969 ± 0.0010	0.3130 ± 0.0010	0.3105 ± 0.0009
	13	0.2943 ± 0.0010	0.3125 ± 0.0010	0.3079 ± 0.0009
	15	0.2943 ± 0.0010	0.3095 ± 0.0010	0.3073 ± 0.0009

Supplementary Table 8: Heterogeneous results in AP, corresponding to Supplementary Figure 12.

Data Type	Hypoxemia	Hypocapnia	Hypotension
raw	0.8866 ± 0.0002	0.8425 ± 0.0002	0.8712 ± 0.0001
ema	0.8916 ± 0.0002	0.8466 ± 0.0002	0.8700 ± 0.0001
auto	0.8909 ± 0.0002	0.8465 ± 0.0002	0.8745 ± 0.0001
next	0.8934 ± 0.0002	0.8528 ± 0.0002	0.8792 ± 0.0001
min	0.8953 ± 0.0002	0.8524 ± 0.0002	0.8806 ± 0.0001
hypo	0.8940 ± 0.0002	0.8551 ± 0.0002	0.8783 ± 0.0001
next′.	0.8922 ± 0.0002	0.8533 ± 0.0002	0.8792 ± 0.0001
min′	0.8938 ± 0.0002	0.8539 ± 0.0002	0.8807 ± 0.0001
hypo′	0.8911 ± 0.0002	0.8491 ± 0.0002	0.8696 ± 0.0001

Supplementary Table 9: Shorter version of performance/transference results for hypo outcomes in ROC AUC, corresponding to Figures 2b-c.

Data Type	Hypoxemia	Hypocapnia	Hypotension
auto	0.2402 ± 0.0014	0.4246 ± 0.0011	0.2974 ± 0.0010
auto′	0.2370 ± 0.0013	0.4228 ± 0.0012	0.3015 ± 0.0010
autonext	0.2509 ± 0.0014	0.4327 ± 0.0012	0.3122 ± 0.0009
autonext′	0.2460 ± 0.0013	0.4397 ± 0.0011	0.3036 ± 0.0010
augauto	0.2366 ± 0.0014	0.3964 ± 0.0011	0.2943 ± 0.0009
augauto′	0.2250 ± 0.0013	0.3969 ± 0.0011	0.2959 ± 0.0010
contrast	0.2368 ± 0.0014	0.4070 ± 0.0012	0.2794 ± 0.0010
contrast′	0.2335 ± 0.0014	0.4047 ± 0.0011	0.2777 ± 0.0009
next	0.2585 ± 0.0014	0.4426 ± 0.0011	0.3116 ± 0.0010
next′	0.2529 ± 0.0013	0.4451 ± 0.0011	0.3123 ± 0.0010

Supplementary Table 10: Additional self supervised results in AP, corresponding to Supplementary Figure 9.

Data Type	Hypoxemia (90)	Hypocapnia (30)	Hypotension (65)
raw	0.1585 ± 0.0014	0.2512 ± 0.0010	0.3967 ± 0.0010
ema	0.1645 ± 0.0014	0.2541 ± 0.0010	0.3873 ± 0.0010
auto	0.1581 ± 0.0014	0.2448 ± 0.0009	0.3925 ± 0.0010
auto’	0.1619 ± 0.0016	0.2499 ± 0.0010	0.3960 ± 0.0009
next	0.1699 ± 0.0015	0.2592 ± 0.0010	0.4138 ± 0.0009
next’	0.1649 ± 0.0014	0.2620 ± 0.0009	0.4109 ± 0.0009
min	0.1800 ± 0.0017	0.2584 ± 0.0009	0.4205 ± 0.0009
min’	0.1712 ± 0.0015	0.2619 ± 0.0009	0.4137 ± 0.0010
hypo	0.1691 ± 0.0015	0.2581 ± 0.0009	0.4052 ± 0.0010
hypo’	0.1675 ± 0.0015	0.2546 ± 0.0010	0.4109 ± 0.0010

Supplementary Table 11: New outcome definition results in AP, corresponding to Supplementary Figure 10

Data Type	Hypotension
raw	0.2626 ± 0.0009
ema	0.2595 ± 0.0009
next	0.2623 ± 0.0009
next′	0.2783 ± 0.0009
next^{ft}	0.2653 ± 0.0009

Supplementary Table 12: Shrink target set results in AP, corresponding to Supplementary Figure 6

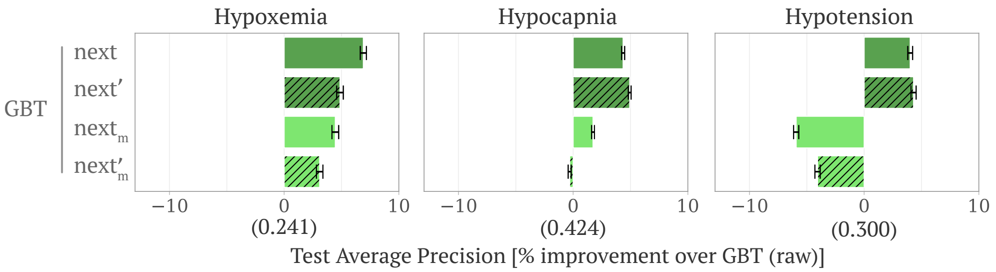

Supplementary Figure 5: *Performance of a jointly trained model.* We train a downstream XGB model to forecast adverse outcomes with different representations of data. First, *next* is the same as in Figure 2. Then, $next_m$ is our jointly trained model. Rather than training each of the 15 LSTMs separately, we train a larger model that jointly trains all 15 networks to forecast the next five minutes of all 15 signals. Finally, (′) denotes transference, as in Figure 2.

We report the performance of XGB trained on these embeddings in Supplementary Figure 5. We observe that $next_m$ performs almost as well as the per-signal *next* models. However, for Hypocapnia and Hypotension, it does not perform nearly as well, even doing worse than *raw* for Hypotension. This could be due to a number of reasons. One possible reason is that multi-task learning is usually beneficial because it increases the effective sample size for one specific outcome using related outcomes; however, in this case we have a large enough sample size to learn the best possible representations. Another possible reason is that the size of the network is much larger in the jointly learned LSTM. With the addition of a loss function that includes many separate signals it may simply constitute a harder optimization problem. As such, we recommend utilizing per-signal embedding models which confer a number of other benefits including using less GPU memory and converging much faster, unless there is a strong reason to do otherwise.

7.1.2 Evaluating by ASA physical status and diagnosis

Given that different surgeries have different outcomes and challenges associated with them, we aim to analyze the performance of our models for a few major surgical diagnoses. We focus on analyzing the performance of XGB models trained with *next* embeddings (from Figure 2). First, we identify the top ten diagnoses for each target operating room dataset according to the distributions in the test set. We evaluate our model's predictions for subsets of samples that correspond to procedures with a given diagnosis. We report the performances for each diagnosis in Supplementary Tables 13-15. Furthermore, because we are comparing across outcomes with different base rates, we report and rely on the results of ROCAUC, rather than average precision (or PRAUC) which is more sensitive to base rates.

We find that for hypoxemia, our model performs strongly across most categories in OR_0 with the exception of "prev c-sect nos-deliver" (repeat cesarean delivery), where hypoxemia is more prevalent. Furthermore, for "cataract" in OR_1, we find that our models are unsuccessful for identifying hypoxemia. For hypocapnia, challenging diagnoses include "senile cataract unspecified" and "cataract". For hypotension, challenging diagnoses include "prev c-sect nos-deliver" and "atrial fibrillation". One possible reason that these particular diagnoses (cataracts and cesareans) were challenging is that they are different to the majority of procedures in the data set. In terms of anesthesia, both are given minimal anesthesia and in the case of cesarean may involve a spinal anesthetic, which has its own characteristic effects. In particular, cesareans and cataracts typically involve regional (97%) or monitored (MAC) (92%) anesthesia respectively in comparison to the 85% of procedures that involve general anesthesia. This finding is in line with recent research in distributionally robust optimization that suggests that even performant models often have poor performance in "minority" or undersampled regions of training data [10] as is the case with MAC and regional anesthesia. Furthermore, they tend to involve only one specific type of patient (cataract = elderly with stiff arteries, cesarean = young and healthy). Finally, the procedures themselves might have their own unique characteristics (cataract = minimal stimulation, cesarean = maximum stimulation) that are on the tails of the normal distribution of physiologic effects. These challenging diagnoses may constitute important future directions of research.

To further analyze differences between procedures, we can stratify the test sets by larger categories that

Target	Diagnosis	# Proc	ROCAUC	PRAUC	BR
OR_0	malign neopl breast nos	66/3807	0.9690 ± 0.0008	0.2843 ± 0.0115	0.85%
	malign neopl breast unspec	54/3807	0.9803 ± 0.0007	0.4778 ± 0.0112	1.20%
	atrial fibrillation	47/3807	0.9360 ± 0.0030	0.3710 ± 0.0136	1.57%
	calculus of kidney	39/3807	0.8769 ± 0.0078	0.2823 ± 0.0175	1.41%
	esophageal reflux	36/3807	0.9787 ± 0.0011	0.5222 ± 0.0166	1.20%
	morbid obesity	32/3807	0.8853 ± 0.0058	0.1328 ± 0.0094	0.83%
	prev c-sect nos-deliver	26/3807	0.7571 ± 0.0101	0.1874 ± 0.0135	2.48%
	malign neopl bladder unspec	26/3807	0.9238 ± 0.0041	0.0943 ± 0.0142	0.40%
	atrial flutter	24/3807	0.9474 ± 0.0033	0.2521 ± 0.0217	0.88%
	malign neoplasm of prostate	23/3807	0.8388 ± 0.0154	0.1640 ± 0.0307	0.19%
OR_1	cataract nos	54/3762	0.7571 ± 0.0108	0.0739 ± 0.0056	1.79%
	calculus of kidney	37/3762	0.8972 ± 0.0043	0.0784 ± 0.0038	1.17%
	internal ortho device complic	34/3762	0.7763 ± 0.0084	0.1148 ± 0.0086	1.83%
	subarachnoid hemorrhage	34/3762	0.8919 ± 0.0027	0.2462 ± 0.0076	2.60%
	senile cataract unspecified	30/3762	0.6731 ± 0.0230	0.2892 ± 0.0299	2.14%
	cataract	28/3762	0.1773 ± 0.0104	0.0082 ± 0.0006	1.17%
	necrotizing fasciitis	26/3762	0.7615 ± 0.0063	0.3608 ± 0.0104	6.01%
	senile cataract nos	26/3762	0.7710 ± 0.0143	0.1359 ± 0.0166	2.35%
	cmp nec d/t orth dev nec	24/3762	0.8302 ± 0.0126	0.2741 ± 0.0191	2.20%
	carpal tunnel syndrome	23/3762	0.5791 ± 0.0122	0.0435 ± 0.0029	3.12%

Supplementary Table 13: XGB with *next* embeddings for subcategories (top 10 diagnoses per target hospital) of the test set. We report the performance for predicting Hypoxemia in ROCAUC, PRAUC (AP), and the base rate of positive examples in each subcategory.

generally represent patient health using ASA physical status (Supplementary Table 16). In general, the most severe codes (V and VI) are very rare, and the results for these categories may be unreliable. For hypoxemia and hypocapnia, we generally find that the more severe the ASA physical status, the higher the base rate. For hypoxemia, hypocapnia, and hypotension, we find that the performance in ROCAUC is generally consistent across the frequently encountered ASA physical statuses (I-IV). Combined with our previous results, we hypothesize that the challenging procedures in terms of forecasting are not the risky procedures, but instead the ones that have very specific patient distributions that are different to the general population.

7.1.3 Evaluating *next* models in a smaller target dataset

In our manuscript, we demonstrated that the fine-tuned embedding model converges faster than the standard embedding model. This assumes that there are two users: one in the target domain who has access to a pre-trained embedding model trained by a user in the source domain. In this setting, we find that it is always better (from the perspective of the user in the target domain) to fine-tune embedding models rather than train them from scratch in terms of both performance and computational requirements. This stands in contrast to the transferred embeddings which greatly reduce computational cost but also slightly decrease performance relative to standard embeddings. A further benefit of the transferred embedding setting is that the user in the target domain is not required to perform expensive hyperparameter searches and can rely on the architecture chosen by an expert who trained embedding models in the source domain.

However, another perspective behind embedding models is their capacity to improve performance in settings where the target dataset is much smaller than the source dataset [16]. To do so, we perform the same experiments from Figures 2 and 3, where we train next embedding models in three ways: (1) standard embedding, (2) transferred embedding, and (3) fine-tuned embedding in target datasets that have roughly 10% the number of procedures that the source dataset does. We report these results in Supplementary Figure 6.

We find that training from scratch is not very beneficial in comparison to *raw* or *ema* when the target training set is so small. This is not surprising given that neural networks often require extremely large data to be effective and suggests that the performance of next in Figure 2 may be contingent on having large training

Target	Diagnosis	# Proc	ROCAUC	PRAUC	BR
OR_0	malign neopl breast nos	66/3807	0.8406 ± 0.0028	0.3515 ± 0.0057	6.09%
	malign neopl breast unspec	54/3807	0.8405 ± 0.0033	0.4626 ± 0.0078	7.97%
	atrial fibrillation	47/3807	0.8178 ± 0.0045	0.4969 ± 0.0095	13.33%
	calculus of kidney	39/3807	0.8083 ± 0.0053	0.5249 ± 0.0095	15.24%
	esophageal reflux	36/3807	0.8722 ± 0.0029	0.3405 ± 0.0082	6.48%
	morbid obesity	32/3807	0.8292 ± 0.0045	0.4203 ± 0.0091	7.89%
	prev c-sect nos-deliver	26/3807	0.0000 ± 0.0000	0.0000 ± 0.0000	0.00%
	malign neopl bladder unspec	26/3807	0.8794 ± 0.0065	0.6035 ± 0.0155	6.66%
	atrial flutter	24/3807	0.8657 ± 0.0062	0.5046 ± 0.0149	8.86%
	malign neoplasm of prostate	23/3807	0.8397 ± 0.0044	0.2638 ± 0.0092	5.38%
OR_1	cataract nos	54/3762	0.8405 ± 0.0060	0.4783 ± 0.0167	12.92%
	calculus of kidney	37/3762	0.8479 ± 0.0050	0.5677 ± 0.0115	9.88%
	internal ortho device complic	34/3762	0.8359 ± 0.0059	0.3781 ± 0.0101	7.69%
	subarachnoid hemorrhage	34/3762	0.8611 ± 0.0043	0.4052 ± 0.0118	10.12%
	senile cataract unspecified	30/3762	0.6689 ± 0.0169	0.4491 ± 0.0256	17.09%
	cataract	28/3762	0.7655 ± 0.0181	0.5406 ± 0.0263	14.29%
	necrotizing fasciitis	26/3762	0.8241 ± 0.0051	0.3501 ± 0.0119	11.82%
	senile cataract nos	26/3762	0.8490 ± 0.0201	0.5494 ± 0.0438	6.52%
	cmp nec d/t orth dev nec	24/3762	0.9677 ± 0.0036	0.7639 ± 0.0178	5.47%
	carpal tunnel syndrome	23/3762	0.8412 ± 0.0081	0.1847 ± 0.0117	5.58%

Supplementary Table 14: XGB with *next* embeddings for subcategories (top 10 diagnoses per target hospital) of the test set. We additionally report the performance in ROCAUC, PRAUC (AP) for predicting Hypocapnia, and the base rate of positive examples in each subcategory.

Target	Diagnosis	# Proc	ROCAUC	PRAUC	BR
OR_0	malign neopl breast nos	66/3807	0.8416 ± 0.0018	0.3164 ± 0.0049	8.01%
	malign neopl breast unspec	54/3807	0.8228 ± 0.0022	0.4596 ± 0.0048	11.87%
	atrial fibrillation	47/3807	0.7897 ± 0.0102	0.2605 ± 0.0173	7.41%
	calculus of kidney	39/3807	0.9122 ± 0.0029	0.4032 ± 0.0136	5.05%
	esophageal reflux	36/3807	0.8995 ± 0.0031	0.4531 ± 0.0112	6.93%
	morbid obesity	32/3807	0.8568 ± 0.0037	0.2127 ± 0.0065	4.38%
	prev c-sect nos-deliver	26/3807	0.7455 ± 0.0050	0.2478 ± 0.0086	10.93%
	malign neopl bladder unspec	26/3807	0.8400 ± 0.0074	0.4555 ± 0.0176	8.70%
	atrial flutter	24/3807	0.8789 ± 0.0032	0.3380 ± 0.0117	4.76%
	malign neoplasm of prostate	23/3807	0.9118 ± 0.0019	0.4003 ± 0.0092	5.65%
OR_1	cataract nos	54/3762	0.9753 ± 0.0029	0.1603 ± 0.0223	0.34%
	calculus of kidney	37/3762	0.9241 ± 0.0035	0.3256 ± 0.0115	3.40%
	internal ortho device complic	34/3762	0.8647 ± 0.0051	0.2870 ± 0.0144	3.20%
	subarachnoid hemorrhage	34/3762	0.8119 ± 0.0050	0.1790 ± 0.0072	6.21%
	senile cataract unspecified	30/3762	0.0000 ± 0.0000	0.0000 ± 0.0000	0.00%
	cataract	28/3762	0.9622 ± 0.0076	0.3709 ± 0.0804	0.27%
	necrotizing fasciitis	26/3762	0.8343 ± 0.0045	0.2728 ± 0.0137	5.23%
	senile cataract nos	26/3762	0.0000 ± 0.0000	0.0000 ± 0.0000	0.00%
	cmp nec d/t orth dev nec	24/3762	0.8510 ± 0.0080	0.5230 ± 0.0183	5.01%
	carpal tunnel syndrome	23/3762	0.9584 ± 0.0027	0.5100 ± 0.0299	2.52%

Supplementary Table 15: XGB with *next* embeddings for subcategories (top 10 diagnoses per target hospital) of the test set. We additionally report the performance in ROCAUC, PRAUC (AP) for predicting Hypotension, and the base rate of positive examples in each subcategory.

Outcome	ASA	Target	# Proc	# Samples	ROCAUC	PRAUC	BR
Hypoxemia	I	OR_0	454/3807	46176/493398	0.9392 ± 0.0019	0.2356 ± 0.0057	0.73%
	I	OR_1	653/3762	94035/559718	0.8293 ± 0.0016	0.1201 ± 0.0025	1.37%
	II	OR_0	1546/3807	186931/493398	0.9211 ± 0.0010	0.2860 ± 0.0030	0.97%
	II	OR_1	1634/3762	230722/559718	0.8564 ± 0.0007	0.1875 ± 0.0018	1.88%
	III	OR_0	1512/3807	216279/493398	0.9236 ± 0.0008	0.3003 ± 0.0029	1.00%
	III	OR_1	1222/3762	195828/559718	0.8533 ± 0.0007	0.2328 ± 0.0015	2.80%
	IV	OR_0	286/3807	43450/493398	0.9258 ± 0.0011	0.3039 ± 0.0043	1.96%
	IV	OR_1	230/3762	36990/559718	0.8673 ± 0.0012	0.3018 ± 0.0029	4.62%
	V	OR_0	9/3807	562/493398	0.9331 ± 0.0040	0.7424 ± 0.0138	12.28%
	V	OR_1	17/3762	1832/559718	0.9253 ± 0.0028	0.6028 ± 0.0115	8.41%
	VI	OR_0	0/3807	0/493398	0.0000 ± 0.0000	0.0000 ± 0.0000	0.00%
	VI	OR_1	4/3762	311/559718	0.7337 ± 0.0109	0.2376 ± 0.0200	8.36%
Hypocapnia	I	OR_0	454/3807	18473/158178	0.8342 ± 0.0014	0.4040 ± 0.0034	8.43%
	I	OR_1	653/3762	47132/232126	0.8821 ± 0.0009	0.4649 ± 0.0025	6.22%
	II	OR_0	1546/3807	66892/158178	0.8336 ± 0.0008	0.3963 ± 0.0018	9.05%
	II	OR_1	1634/3762	97475/232126	0.8601 ± 0.0006	0.4586 ± 0.0015	7.90%
	III	OR_0	1512/3807	62470/158178	0.8491 ± 0.0007	0.4654 ± 0.0020	10.89%
	III	OR_1	1222/3762	75330/232126	0.8593 ± 0.0006	0.4410 ± 0.0017	8.86%
	IV	OR_0	286/3807	10222/158178	0.8411 ± 0.0014	0.4642 ± 0.0039	12.02%
	IV	OR_1	230/3762	12023/232126	0.8435 ± 0.0018	0.4462 ± 0.0041	9.18%
	V	OR_0	9/3807	121/158178	0.7291 ± 0.0146	0.5402 ± 0.0238	21.49%
	V	OR_1	17/3762	165/232126	1.0000 ± 0.0000	1.0000 ± 0.0000	7.88%
	VI	OR_0	0/3807	0/158178	0.0000 ± 0.0000	0.0000 ± 0.0000	0.00%
	VI	OR_1	4/3762	1/232126	0.0000 ± 0.0000	0.0000 ± 0.0000	100.00%
Hypotension	I	OR_0	454/3807	27471/234659	0.8748 ± 0.0009	0.3558 ± 0.0029	7.15%
	I	OR_1	653/3762	66482/320067	0.8944 ± 0.0007	0.2809 ± 0.0024	3.42%
	II	OR_0	1546/3807	108207/234659	0.8638 ± 0.0005	0.3462 ± 0.0014	7.47%
	II	OR_1	1634/3762	148732/320067	0.8890 ± 0.0005	0.2374 ± 0.0016	3.21%
	III	OR_0	1512/3807	88423/234659	0.8740 ± 0.0005	0.3911 ± 0.0017	7.49%
	III	OR_1	1222/3762	93663/320067	0.8878 ± 0.0006	0.2739 ± 0.0017	3.95%
	IV	OR_0	286/3807	10528/234659	0.8426 ± 0.0019	0.3097 ± 0.0045	6.89%
	IV	OR_1	230/3762	10520/320067	0.8866 ± 0.0019	0.3085 ± 0.0051	4.87%
	V	OR_0	9/3807	30/234659	0.0000 ± 0.0000	0.0000 ± 0.0000	0.00%
	V	OR_1	17/3762	551/320067	0.9833 ± 0.0014	0.6370 ± 0.0276	3.45%
	VI	OR_0	0/3807	0/234659	0.0000 ± 0.0000	0.0000 ± 0.0000	0.00%
	VI	OR_1	4/3762	119/320067	0.0000 ± 0.0000	0.0000 ± 0.0000	0.00%

Supplementary Table 16: XGB with *next* embeddings for subcategories (ASA physical statuss) of the test set. We additionally report the performance in ROCAUC, PRAUC (AP), and the base rate of positive examples in each subcategory.

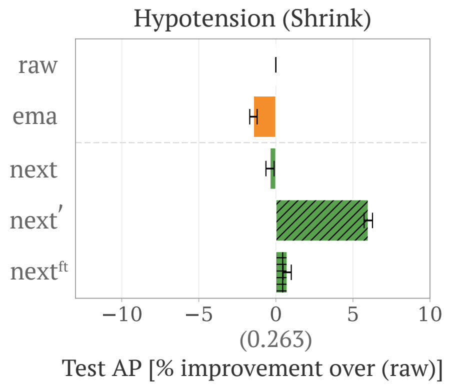

Supplementary Figure 6: *Performance on a smaller target set (10%).* We perform the same experiments from Figures 2 and 3, where we train next embedding models in three ways: (1) standard embedding, (2) transferred embedding, and (3) fine-tuned embedding. However, when we perform the experiments, we first greatly subsample the target training dataset to 3000 procedures (roughly 10% of each target dataset's full sample size). This simulates the setting where the target hospital is much smaller than the source hospital.

data sets. We also find that the performance of next' is quite high, suggesting that the transferred embedding model from a much larger data set is the best approach in this setting. Finally, we find that fine tuning ($next^{ft}$) does consistently improve over the standard embedding setting (*next*), but the improvements are small relative to the transferred embedding approach (*next*'). This suggests that the fine-tuned embedding models find similar local minima to the standard embedding models that are simply not as performant due to the low sample size of the target data set. Although fine tuned embedding models were not the best approach in this low sample size setting, it is important to note that they may still be advisable under dataset drift. For our experiment we utilized a random subsample of the target dataset, which we found to be similar in distribution to the source dataset. However, if the target user suspects that their target domain is very different to the source domain, fine-tuning is likely the most appropriate approach.

7.1.4 Evaluating additional self-supervised approaches

There are a wide variety of self-supervised learning techniques that may be applicable in our experimental setting. We review three broad categories here: (1) methods that incorporate future knowledge, (2) data augmentation approaches, and (3) contrastive learning approaches.

There are several approaches that incorporate the future into self-supervised training. In fact, one of the earliest applications of self-supervised learning involves LSTM networks to learn representations of video sequences [20]. Srivastava, Mansimov, and Salakhudinov [20] utilizes three pre-training tasks: (1) predicting the current frames, (2) predicting the future frames, and (3) two decoders predicting the current and future frames simultaneously. In a different domain, Devlin et al. [6] generates natural language representations by pre-training with two tasks: (1) predicting randomly masked input tokens and (2) predicting next sentences. Both papers utilize an analogous task to our *next* task for different domains and predicting the current frames as in Srivastava, Mansimov, and Salakhudinov [20] is analogous to our *auto* task. In a more related domain, Spathis et al. [19] learn representations of activity signals (accelerometers) by forecasting heart rates. This is similar to *next*; however, Spathis et al. [19]'s outcome is drawn from a different signal (electrocardiogram) to the input signal (accelerometer).

Another broad category of self-supervised approaches has been applied in the domain of human activity recognition and focuses on data augmentation. In particular, Saeed, Ozcelebi, and Lukkien [18] and Tang et al. [21] utilize data augmentations to generate self-supervised labels. Both approaches apply eight transformations on accelerometer data. Saeed, Ozcelebi, and Lukkien [18] focuses on classifying whether one of the eight transformations was applied to the input signal. Tang et al. [21] builds upon this self-supervised approach using teacher-student learning.

A final major category of self-supervised approaches fall under the broad category of contrastive learning.

The high level goal of contrastive learning is to learn representations that group together similar images (positive pairs) and push apart dissimilar images (negative pairs). Chen et al. [4] and Grill et al. [9] propose self-supervised contrastive learning approaches for video representations. Kiyasseh, Zhu, and Clifton [11] and Banville et al. [1] apply contrastive learning for cardiac and electroencephalography signals respectively. Contrastive learning typically consists of the following components: (1) stochastic data augmentation to generate positive pairs, (2) a base encoder that extracts representation vectors, (3) a projection head that maps representations to space for the contrastive loss, and (4) a contrastive loss function that tries to identify pairs within sets of samples.

All of the aforementioned categories of self-supervised approaches have a large number of parameters. We will focus on comparing to a representative method from each category:

- *autonext* - follows the form of Srivastava, Mansimov, and Salakhudinov [20] which trains LSTM embedding models using both the future and the past signals. In this outcome we train the same LSTMs from before but predict both the auto (t-60:t) and (t:t+5) next outcomes (t-60:t+5).

- *augauto* - utilizes the augmentation techniques utilized in Saeed, Ozcelebi, and Lukkien [18] and Tang et al. [21] and trains the LSTM embedding models to predict whether one of seven transformations of the original signal were performed: noised, scaled, rotated, negated, horizontal flip, permute, time-warp. We exclude the final transformation (channel shuffling) because it is not applicable to our signals. We visualize the augmentations in Supplementary Figure 7.

- *contrast* - follows the contrastive learning approaches in Chen et al. [4] and Grill et al. [9] and trains a siamese network to predict positive pairs of signal data using the contrastive loss from SimCLR [4]. We perform the transformations from the augauto approach in order to generate positive pairs of samples. We can verify that in comparison to *auto*, the *contrast* approach we employ does indeed distinguish positive and negative pairs of samples in Supplementary Figure 8.

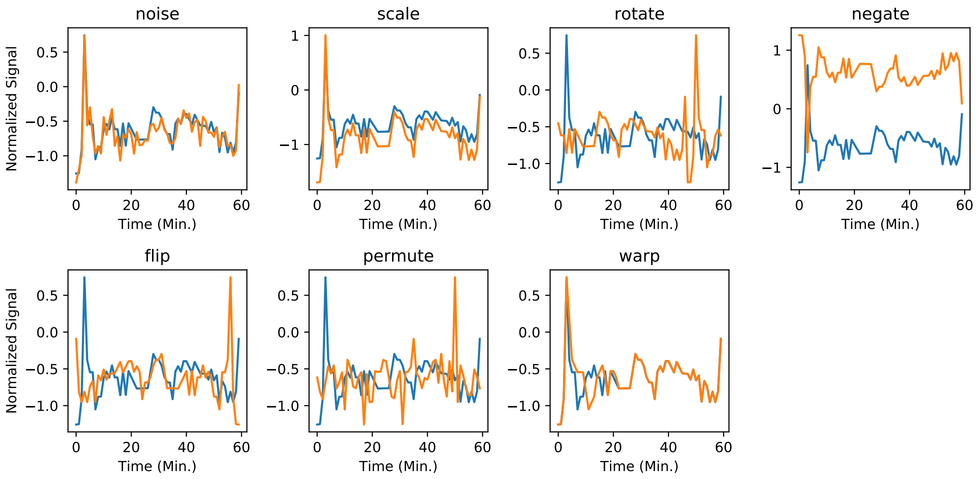

Supplementary Figure 7: Samples of the data augmentations we perform.

In Supplementary Figure 9, we find that compared to our previous approaches, the augmentation-based approaches (*augauto* and *contrast*) do not appear to improve performance, and actually appear to be destructive compared to the straightforward *auto* task. One possible reason is that these approaches are likely heavily dependent on the augmentations we apply and would require a great deal of hyperparameter searching to identify the most appropriate augmentations. Finally, *autonext* appears to improve performance relative to *auto*, but is consistently worse than *next*. Put together, these results suggest that these additional self-supervised approaches fail to improve performance beyond our straightforward *next* approach for the signals and outcomes we consider. Furthermore, future work on data augmentation techniques or contrastive learning that incorporates future signals may be valuable as well.

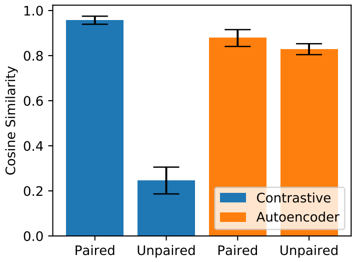

Supplementary Figure 8: Cosine similarity of positive and negative pairs of samples for *contrast* and *auto* embeddings.

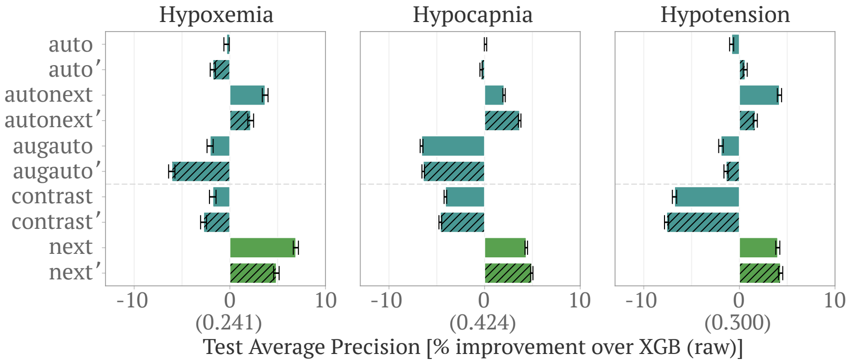

Supplementary Figure 9: XGB models trained with embedded versions of the fifteen physiological signals concatenated with static features (analogous to Figure 2). We report the absolute performance of XGB *raw* below 0 on the x axis in parenthesis.

7.1.5 Evaluating alternative outcome definitions

There are many possible thresholds that have been defined with respect to hypoxemia, hypocapnia, and hypotension. Importantly, the goal of PHASE is not to identify the best definition of each outcome, but rather to evaluate our self-supervised approach on a variety of diverse outcomes. However, to show that our models are relatively robust to choice of threshold for our outcomes, we include analysis of our models from Figure 2 by evaluating three new outcomes: SAO2$\leq$ 90%, ETCO2<30 mm Hg, and NIBPM<65 mm Hg in Supplementary Figure 10. In general, we find that our *next* and *min* embeddings are consistently beneficial for the new outcome definitions. For hypoxemia (90), the *min* models are particularly beneficial, perhaps because the base rate of hypoxemia at this threshold is much lower.

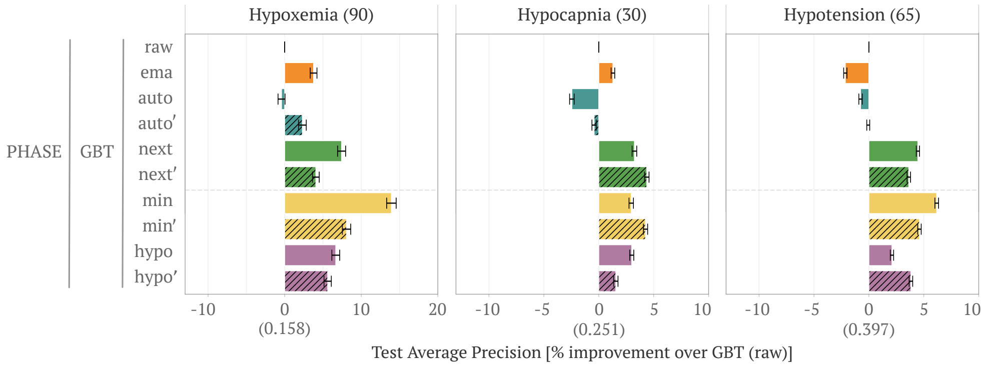

Supplementary Figure 10: Analyses analogous to Figure 2 for hypoxemia, hypocapnia, and hypotension at different thresholds. Here we consider SAO2 < 90 for hypoxemia, ETCO2 < 30 for hypocapnia, and NIBPM < 65 for hypotension.

7.2 MLP downstream model

One potential criticism of our evaluations in previous are that we evaluate with only one downstream model type: XGB. There are clearly a number of benefits to using tree-based methods such as ease of training, exact SHAP value attribution methods, performance on par with LSTMs for our datasets, and more. However, in order to show that *PHASE embeddings improve performance and transference for a variety of downstream model types*, we replicate Figures 2b and 2c using MLP downstream models in Supplementary Figure 11. We see that, as with downstream XGB models, the PHASE embeddings offer a substantial improvement over *raw* embeddings for downstream MLP models.

We utilize multi-layer perceptrons as our downstream model. We train the MLPs with embedding features from 15 physiological signals, resulting in a total of 3000 features for PHASE methods. In addition, we concatenate static features to the signal features to train and evaluate the models. The model's architecture consists of the following: a dense layer with 100 nodes (with a relu activation) followed by a dropout layer with dropout rate 0.5 followed by a dense layer with 100 nodes (with a relu activation) followed by a dropout layer with dropout rate 0.5 followed by the dense output layer with one node and sigmoid activation function. We utilize a learning rate of 0.00001, adam optimizer, and binary cross entropy loss. We found that 200 epochs was sufficient for the downstream models to converge. We fix hyperparameter settings across experiments so that we can focus on comparing different representations our signal data. In order to train these models, we utilize 72 CPUs (Intel(R) Xeon(R) CPU E5-2699 v3 @ 2.30GHz)

7.3 Applying PHASE for heterogeneous features

In this section, we aim to evaluate PHASE for heterogeneous feature sets by acting as if the target data has fewer physiological signals than it actually does. To do so, we impose an ordering on the features according to the importance of each signal for the XGB model trained on *raw* data (Figures 13b for hypoxemia, 14b for hypocapnia, 15b for hypotension). Then, we run XGB to forecast our outcomes with increasing subsets of

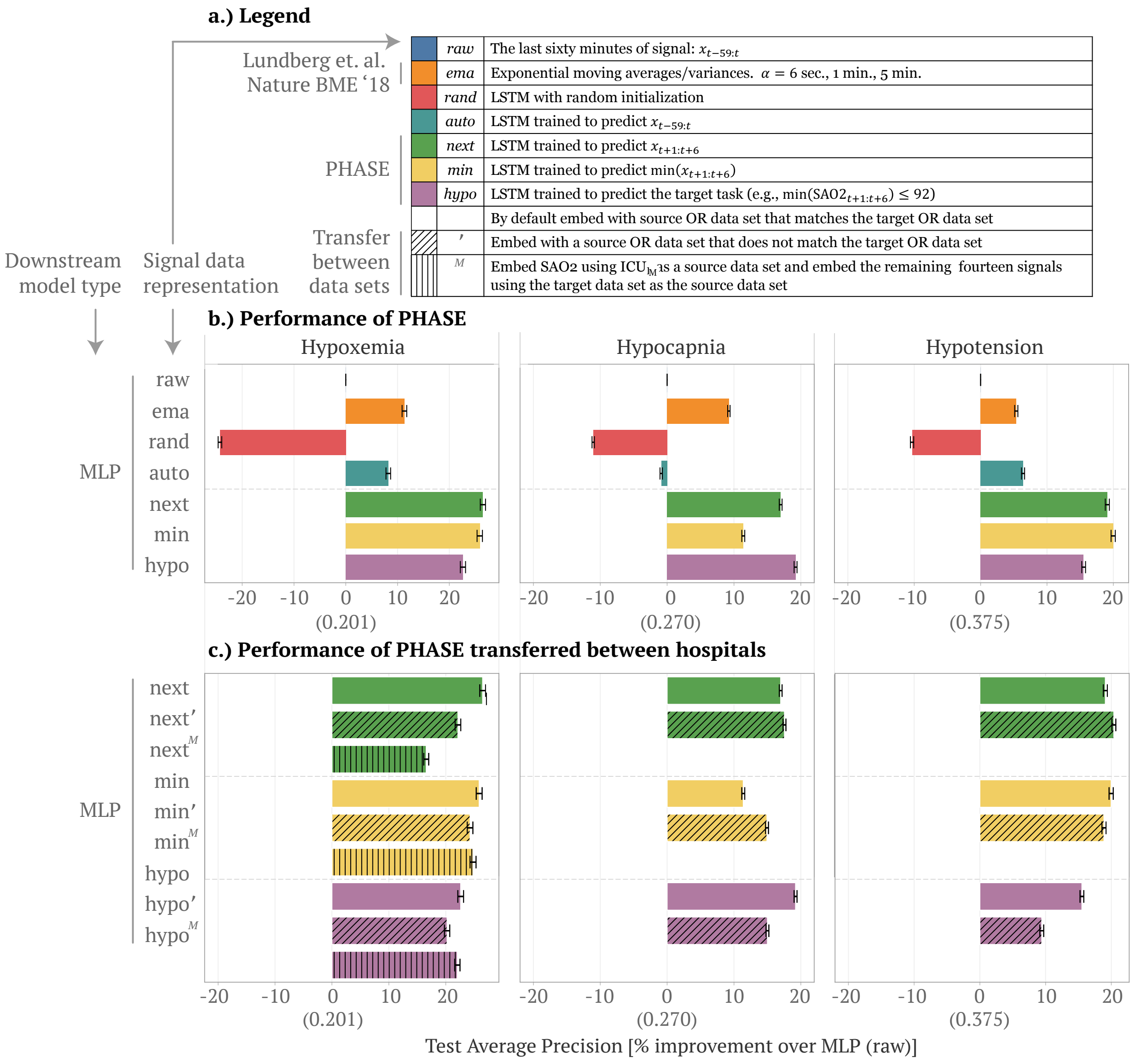

Supplementary Figure 11: Performance of PHASE embeddings with MLP downstream model rather than XGB as in Figure 2. We report the average precision value of the *raw* model in parenthesis on the x-axis.

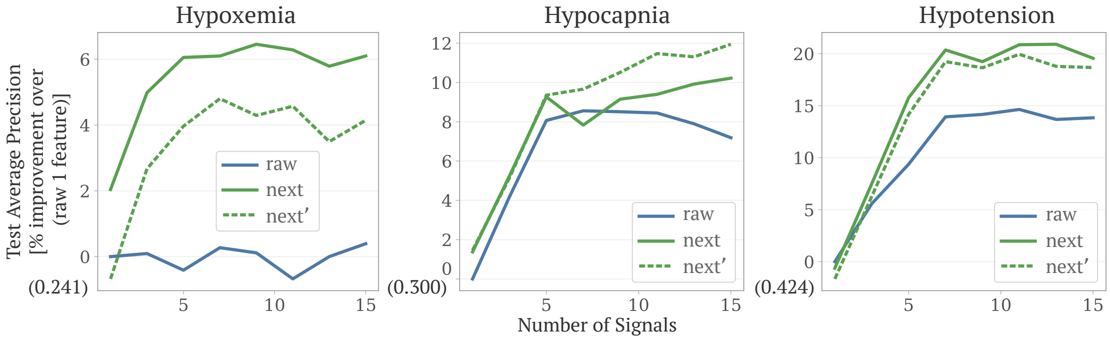

Supplementary Figure 12: *Heterogeneous features.* Performance of PHASE for XGB trained on subsets of the features on the target data. For these models we do not concatenate the six static features and instead focus on the signal data exclusively. We report the average precision value of the *raw* model in parenthesis on the x-axis.

features by including the most important features first. In Supplementary Figure 12, we can see that for all three outcomes, *next* and *next′*, are either comparable to the raw representation or significantly better. One interesting relationship is that the improvement over the *raw* signal becomes greater with an increased number of signals. This implies that the embeddings allow the downstream model to better use signals in conjunction, whereas the embedding of the most important feature for each task is not as useful on its own.

7.4 Full summary plots

In this section, we show the full summary plots (Supplementary Figures 13-17) for the per-feature and aggregated attributions for *raw* and *next* models trained from XGB models trained in target dataset OR_0. We can see more relationships between each of the five downstream tasks and the top 20 features sorted by the mean absolute SHAP values for each feature.

7.5 Evaluating different embedding sizes

In the majority of experiments we evaluate PHASE using LSTM hidden state embeddings of size 200. We chose size 200 because embeddings of this size were performant; however, we did not go beyond 200 because we were constrained by memory. In order to train XGB models, we need to load the entire training dataframe into memory, which requires an amount of memory that is multiplicatively dependent on the embedding size: O(# Samples * (Embedding Size * # Physiological Features + # Static Features). However, the model architecture can still influence the efficacy of the resultant embeddings. In order to examine the impact of embedding size on the downstream predictive performance, we re-trained the *next* LSTM models for all physiological signals with identical model architectures as described in Supplementary Section 6.1, except with different numbers of LSTM nodes in the second (final) hidden layer. In Supplementary Figure 18, we see that the performance of XGB models does indeed improve with larger embedding sizes. It also appears that the performance of the XGB models begins to saturate around embedding sizes of 200; however, it may be possible to further improve performance by increasing embedding sizes further. Although increasing embedding size may improve downstream predictive performance (with decreasing marginal returns), we find that doing so is more computationally expensive in terms of both memory and time. Future work could involve training maximally informative embeddings that simultaneously minimize embedding size.

7.6 Evaluating embedding time slices

LSTM models operate by unrolling backpropagation for a number of time steps. This means that we can obtain embeddings of hidden states for a different number of time steps, rather than just for the final time step (as is the default behavior in Keras). In order to evaluate the impact of returning multiple time steps of

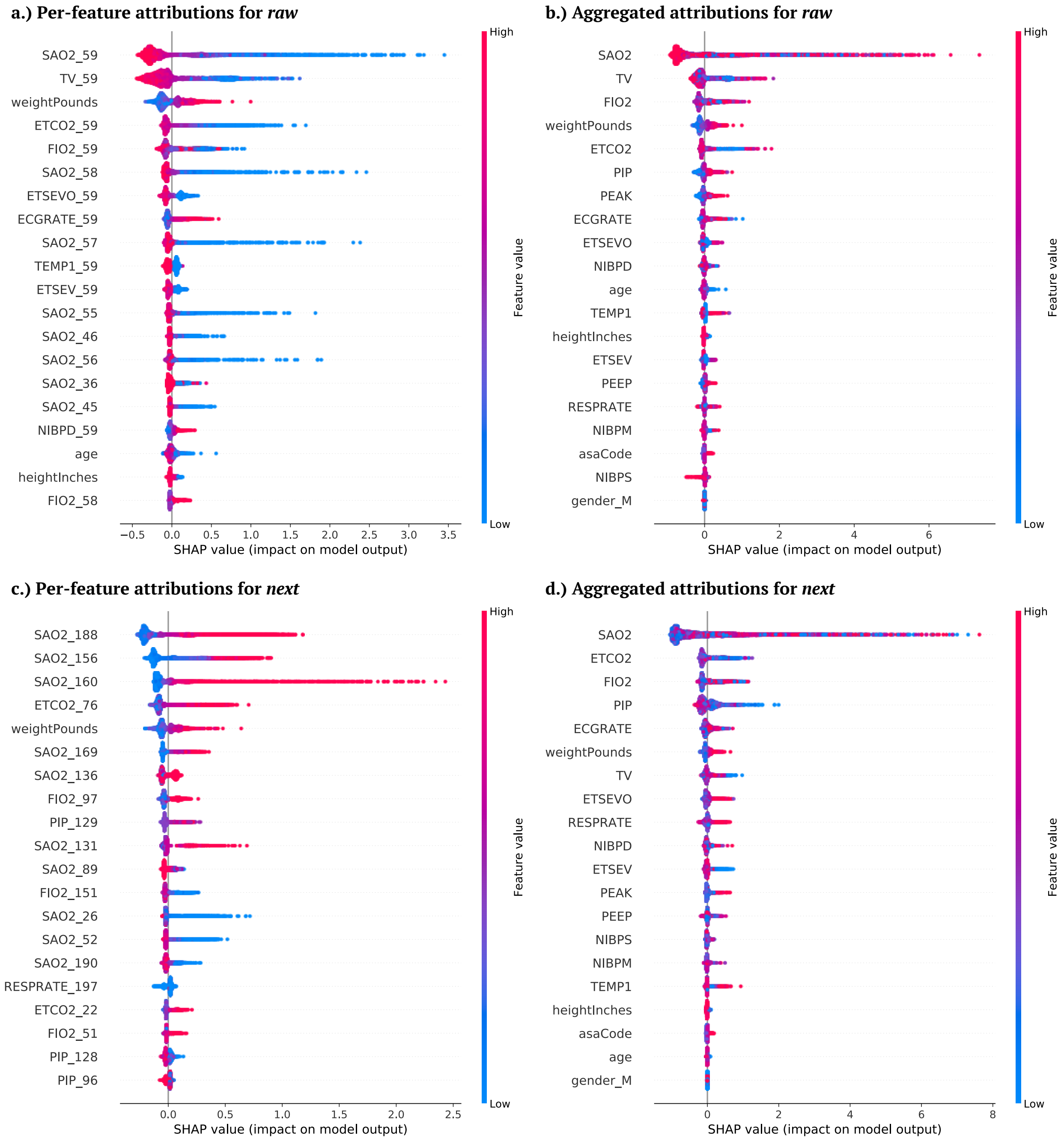

Supplementary Figure 13: Attributions for hypoxemia.

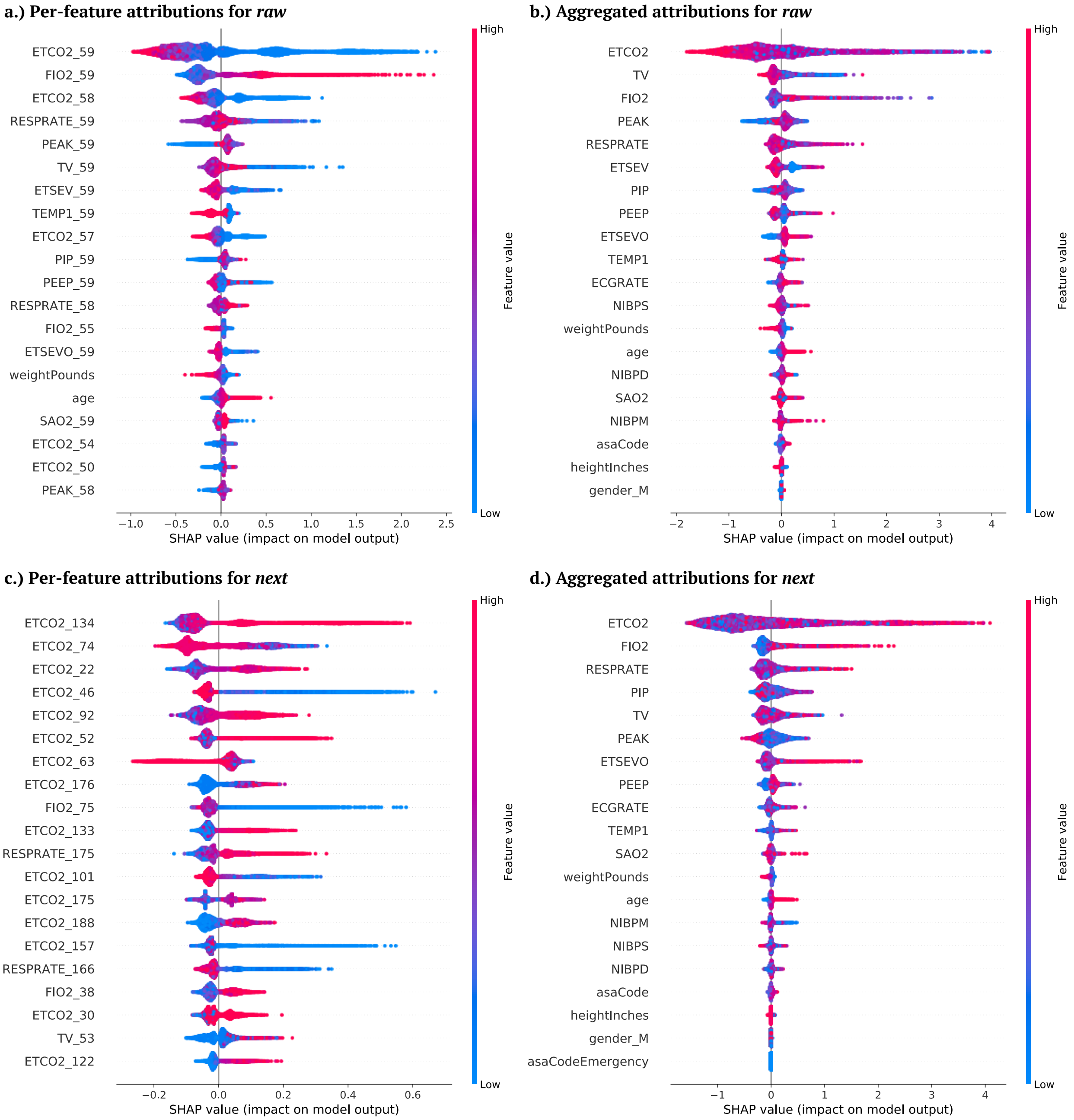

Supplementary Figure 14: Attributions for *hypocapnia*.

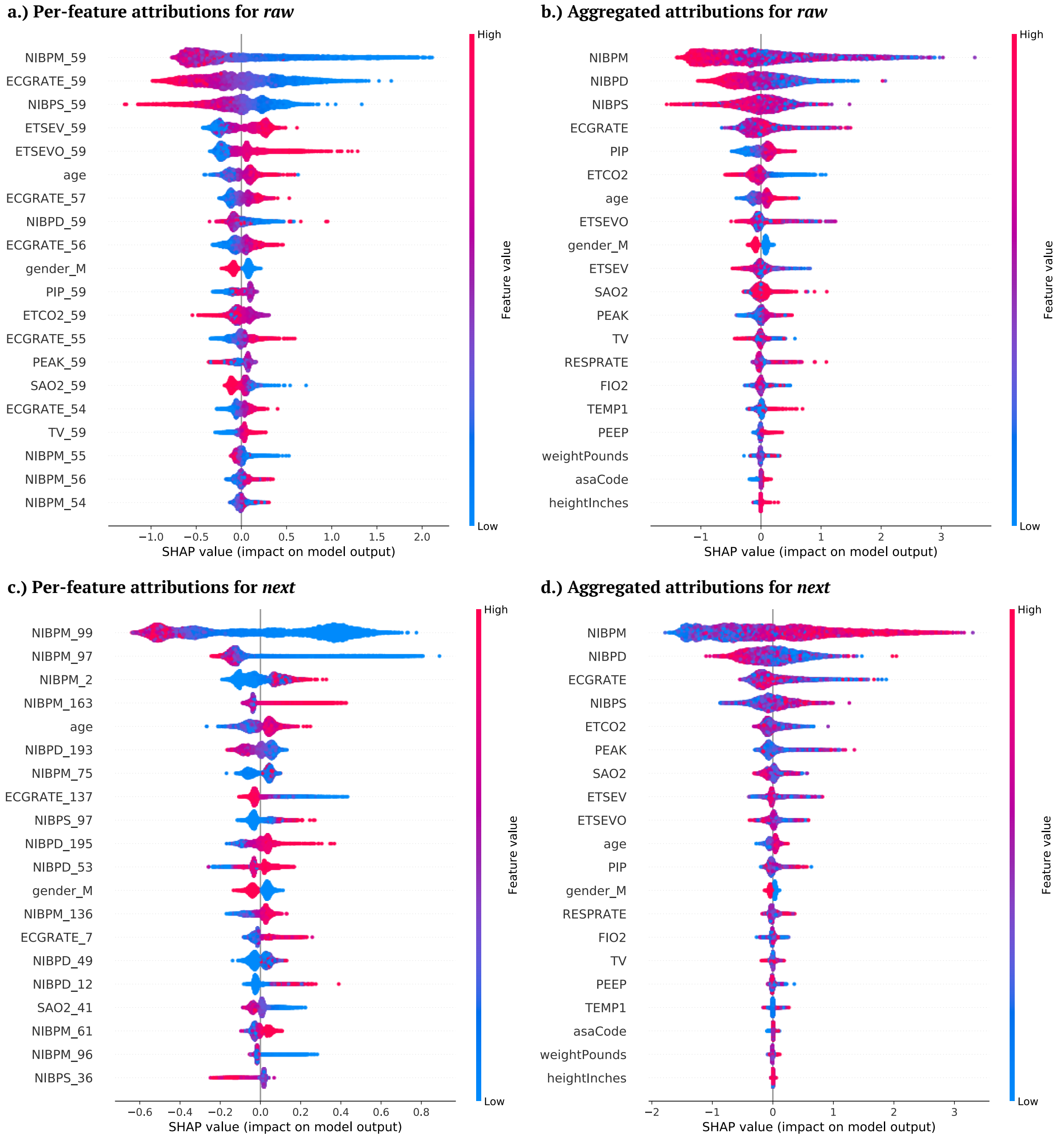

Supplementary Figure 15: Attributions for *hypotension*.

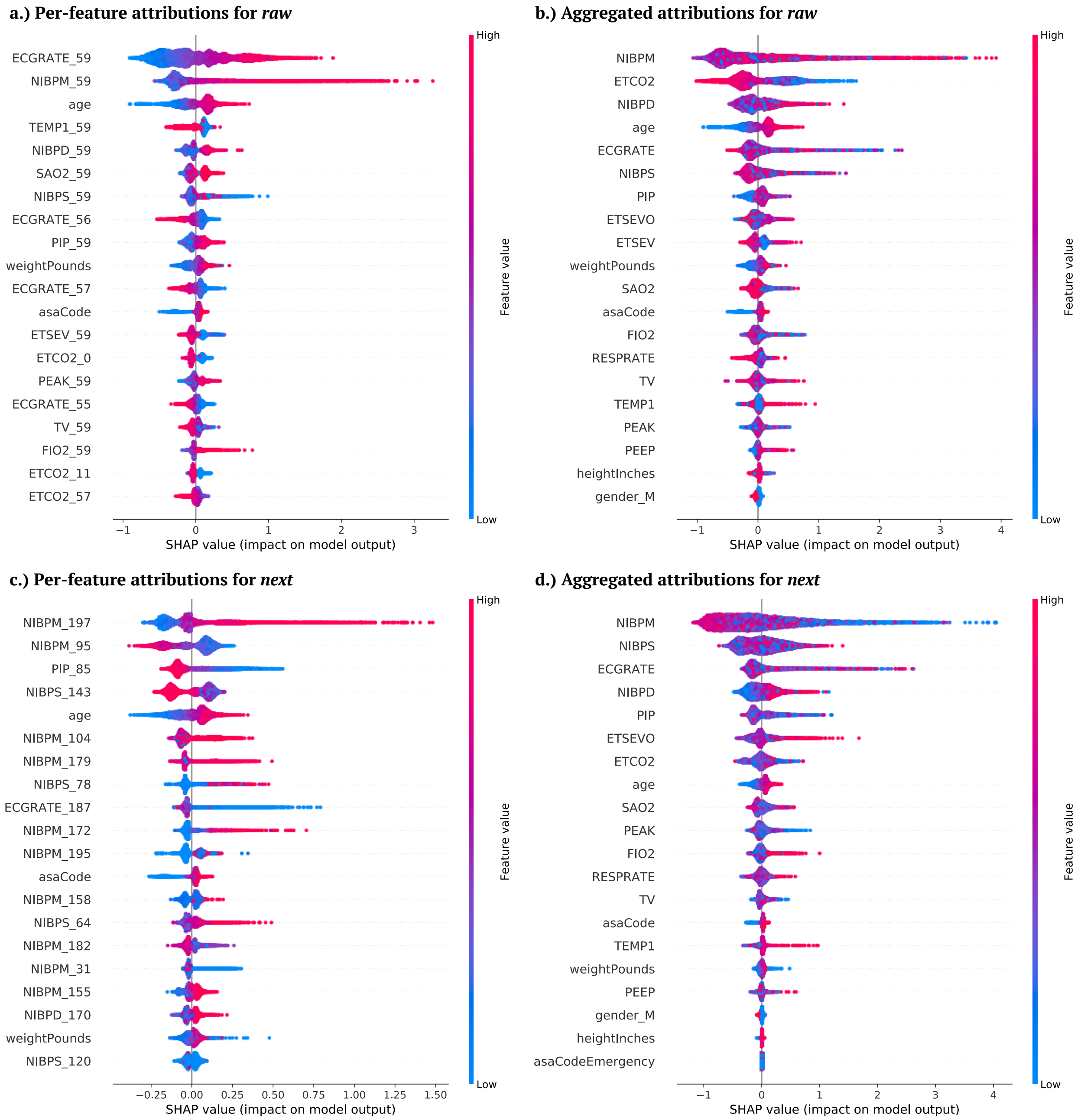

Supplementary Figure 16: Attributions for *hypertension*.

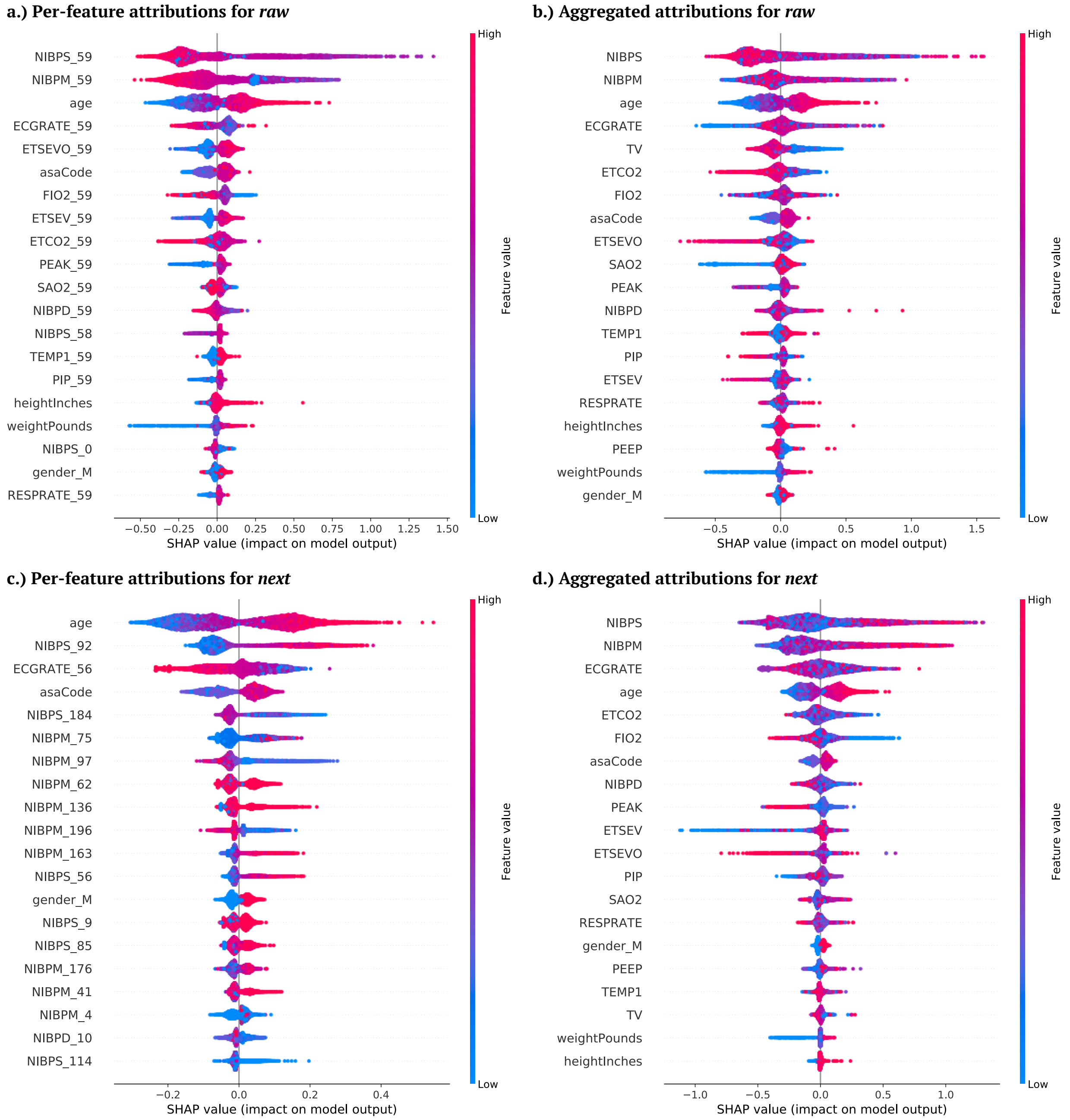

Supplementary Figure 17: Attributions for *phenylephrine*.

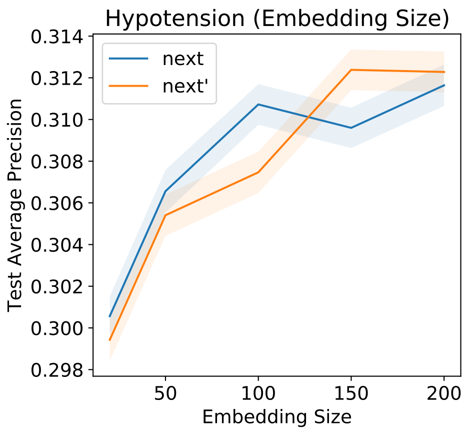

Supplementary Figure 18: *Comparison of different embedding sizes.* We train XGB models on *next* LSTM embedded data (15 embedded signals and 6 static features) using different embedding sizes. We focus on predicting hypotension which has fewer samples than alternative outcomes. The shaded regions show 99% confidence intervals.

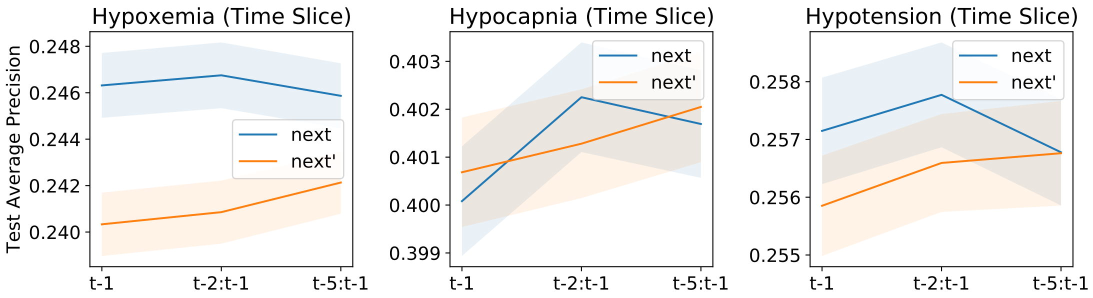

Supplementary Figure 19: *Comparison of embedding time slices.* We train XGB models on a single embedded feature (SAO2 for hypoxemia, ETCO2 for hypocapnia, and NIBPM for hypotension). The y axis represents the test average precision of the XGB model and the x axis represents the time slice we utilize for the final embedding. $t-1$ corresponds to the embedding for the most recent time point (size 200), $t-2:t-1$ corresponds to the embedding for the two most recent time points (size 400), and $t-5:t-1$ corresponds to the embedding for the five most recent time points (size 1000). The shaded regions show 99% confidence intervals.

the final embedding, we include more time steps (slices) for a single feature for our three hypo outcomes in Supplementary Figure 19.

We focus on analyzing the effect of time slices for a single physiological feature's embedding because our XGBoost models with all fifteen physiological feature's already consume upwards of 300GB of memory for the hypoxemia outcome. Including multiple time slices would multiplicatively increase this, with five time slices constituting more than a terabyte of memory. In Supplementary Figure 19, we see that increasing the number of time slices can improve performance for the transferred models (*next'*), but overall the marginal improvement in performance from incorporating the information from more time slices is relatively small. Furthermore, for the non-transferred models (*next*), we do not find that more time slices yields better performance. As such, utilizing the final time step's embedding as we have done throughout our experiments seems to be the best approach.

7.7 Evaluating window size

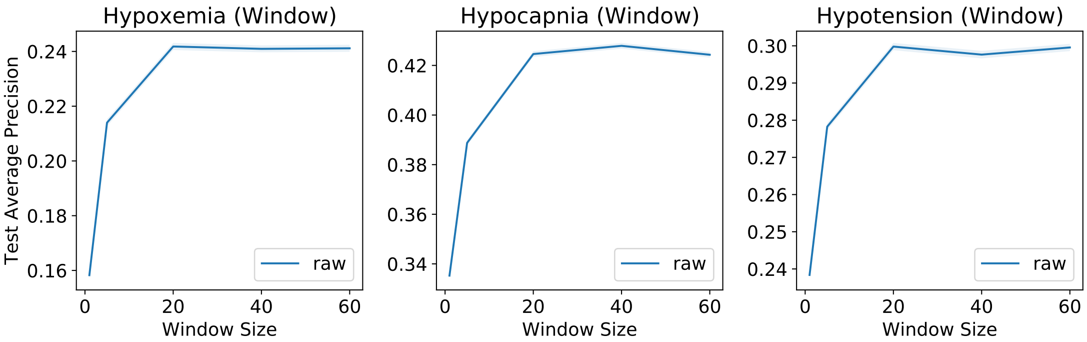

Supplementary Figure 20: *Comparison of window sizes.* We train *raw* XGB models on all fifteen physiological features and static features. We use varying window sizes for the physiological signals and report test average precision.

The window size we use in our experiments is 60 minutes which amounts to 60 features from each signal, because they are sampled minute by minute. However, the window size is actually an additional hyperparameter. The primary motivation to use 60 minutes is twofold: (1) an hour is an easy to understand choice of hyperparameter and (2) we found that 60 minutes was sufficient to make the best possible predictions. In particular, we can evaluate (2) by training XGB models with raw data that includes different window sizes: 60 minutes (as is the default choice in our paper), 40 minutes, 20 minutes, 5 minutes, and 1 minutes. In Supplementary Figure 20, we find that at around 20 minutes we are mostly saturated in terms of the useful information to predict our outcomes.

References

[1] Hubert Banville et al. "Self-supervised representation learning from electroencephalography signals". In: *2019 IEEE 29th International Workshop on Machine Learning for Signal Processing (MLSP)*. IEEE. 2019, pp. 1–6.

[2] Kenneth M Brady et al. "Personalizing the definition of hypotension to protect the brain". In: *Anesthesiology* 132.1 (2020), pp. 170–179.

[3] Tianqi Chen and Carlos Guestrin. "Xgboost: A scalable tree boosting system". In: *Proceedings of the 22nd acm sigkdd international conference on knowledge discovery and data mining*. 2016, pp. 785–794.

[4] Ting Chen et al. "A simple framework for contrastive learning of visual representations". In: *International conference on machine learning*. PMLR. 2020, pp. 1597–1607.

[5] Francois Chollet et al. *Keras*. 2015. URL: `https://github.com/fchollet/keras`.

[6] Jacob Devlin et al. "Bert: Pre-training of deep bidirectional transformers for language understanding". In: *arXiv preprint arXiv:1810.04805* (2018).

[7] Philippe Dony, Michele Dramaix, and Jean G Boogaerts. "Hypocapnia measured by end-tidal carbon dioxide tension during anesthesia is associated with increased 30-day mortality rate". In: *Journal of clinical anesthesia* 36 (2017), pp. 123–126.

[8] Felix A. Gers, Jurgen Schmidhuber, and Fred Cummins. "Learning to forget: Continual prediction with LSTM". In: *Neural Computation* 12.10 (2000), pp. 2451–2471.

[9] Jean-Bastien Grill et al. "Bootstrap your own latent: A new approach to self-supervised learning". In: *arXiv preprint arXiv:2006.07733* (2020).

[10] Tatsunori Hashimoto et al. "Fairness without demographics in repeated loss minimization". In: *International Conference on Machine Learning*. PMLR. 2018, pp. 1929–1938.

[11] Dani Kiyasseh, Tingting Zhu, and David A Clifton. "Clocs: Contrastive learning of cardiac signals". In: *arXiv preprint arXiv:2005.13249* (2020).

[12] Scott M Lundberg et al. "Explainable machine-learning predictions for the prevention of hypoxaemia during surgery". In: *Nature biomedical engineering* 2.10 (2018), pp. 749–760.

[13] Sumit R Majumdar et al. "Oxygen saturations less than 92% are associated with major adverse events in outpatients with pneumonia: a population-based cohort study". In: *Clinical infectious diseases* 52.3 (2011), pp. 325–331.

[14] Martin Abadi et al. *TensorFlow: Large-Scale Machine Learning on Heterogeneous Systems*. Software available from tensorflow.org. 2015. URL: `https://www.tensorflow.org/`.

[15] World Health Organization et al. "Pulse oximetry training manual". In: *Geneva: World Health Organization* (2011).

[16] Yeping Lina Qiu et al. "A meta-learning approach for genomic survival analysis". In: *Nature communications* 11.1 (2020), pp. 1–11.

[17] David L Reich et al. "Predictors of hypotension after induction of general anesthesia". In: *Anesthesia & Analgesia* 101.3 (2005), pp. 622–628.

[18] Aaqib Saeed, Tanir Ozcelebi, and Johan Lukkien. "Multi-task self-supervised learning for human activity detection". In: *Proceedings of the ACM on Interactive, Mobile, Wearable and Ubiquitous Technologies* 3.2 (2019), pp. 1–30.

[19] Dimitris Spathis et al. "Self-supervised transfer learning of physiological representations from free-living wearable data". In: *Proceedings of the Conference on Health, Inference, and Learning*. 2021, pp. 69–78.

[20] Nitish Srivastava, Elman Mansimov, and Ruslan Salakhudinov. "Unsupervised learning of video representations using lstms". In: *International conference on machine learning*. PMLR. 2015, pp. 843–852.

[21] Chi Ian Tang et al. "SelfHAR: Improving Human Activity Recognition through Self-training with Unlabeled Data". In: *arXiv preprint arXiv:2102.06073* (2021).

[22] Michael Walsh et al. “Relationship between intraoperative mean arterial pressure and clinical outcomes after noncardiac surgery: toward an empirical definition of hypotension”. In: *Anesthesiology* 119.3 (2013), pp. 507–515.

[23] Megan Way and Gary E Hill. “Intraoperative end-tidal carbon dioxide concentrations: what is the target?” In: *Anesthesiology research and practice* 2011 (2011).
