# Supplementary material for: Forecasting adverse surgical events using self-supervised transfer learning for physiological signals
Source: NPJ Digit Med. 2021 Dec 8;4:167. doi: 10.1038/s41746-021-00536-y (PMC8654960; doi:10.1038/s41746-021-00536-y)
Supplement: Supplementary file 2 — Reporting Summary [file 41746_2021_536_MOESM2_ESM.pdf]

nature portfolio

Corresponding author(s): Su-In Lee
Last updated by author(s): Jun 8, 2021

Reporting Summary

Nature Portfolio wishes to improve the reproducibility of the work that we publish. This form provides structure for consistency and transparency in reporting. For further information on Nature Portfolio policies, see our Editorial Policies and the Editorial Policy Checklist.

Statistics

For all statistical analyses, confirm that the following items are present in the figure legend, table legend, main text, or Methods section.

n/a	Confirmed	
☐	☒	The exact sample size (*n*) for each experimental group/condition, given as a discrete number and unit of measurement
☐	☒	A statement on whether measurements were taken from distinct samples or whether the same sample was measured repeatedly
☐	☒	The statistical test(s) used AND whether they are one- or two-sided<br>*Only common tests should be described solely by name; describe more complex techniques in the Methods section.*
☐	☒	A description of all covariates tested
☒	☐	A description of any assumptions or corrections, such as tests of normality and adjustment for multiple comparisons
☒	☐	A full description of the statistical parameters including central tendency (e.g. means) or other basic estimates (e.g. regression coefficient) AND variation (e.g. standard deviation) or associated estimates of uncertainty (e.g. confidence intervals)
☒	☐	For null hypothesis testing, the test statistic (e.g. *F*, *t*, *r*) with confidence intervals, effect sizes, degrees of freedom and *P* value noted<br>*Give P values as exact values whenever suitable.*
☒	☐	For Bayesian analysis, information on the choice of priors and Markov chain Monte Carlo settings
☒	☐	For hierarchical and complex designs, identification of the appropriate level for tests and full reporting of outcomes
☒	☐	Estimates of effect sizes (e.g. Cohen's *d*, Pearson's *r*), indicating how they were calculated

Our web collection on statistics for biologists contains articles on many of the points above.

Software and code

Policy information about availability of computer code

Data collection	The OR hospital systems utilize AIMS (Merge AIM, Merge Inc.), a system that automatically captures minute-by-minute hemodynamic and ventilation parameters from the patient monitor and the anaesthesia machine. The system integrates with other hospital EHR systems to automatically acquire laboratory and patient registration information. The automatic data collection is supplemented by manual documentation of medications and anaesthesia interventions to form the complete anaesthesia record during a surgery. For this project, we extracted the high-fidelity anaesthesia data from the AIMS database from May 2012 to June 2014. The medical history data of each patient were also extracted from the hospital systems' EHR data warehouse (Caradigm). The anaesthesia record data and the corresponding medical history data constitute the data we used to evaluate PHASE.<br><br>The ICU data is from the MIMIC-III database populated by data acquired during routine hospital care from sources including archives from critical care information systems, hospital EHR database, and the Social Security Administration Death Master file. They utilized two critical care information systems: Philips CareVue Clinical Information System (models M2331A and M1215A; Philips Health-care, Andover, MA) and iMDsoft MetaVision ICU (iMDsoft, Needham, MA) which measure time-stamped nurse-verified physiological measurements and more.
Data analysis	Deep models were trained using Python 3.6.8, Tensorflow 1.14.0, Keras 2.2.4, NVIDIA GeForce GTX 1080 Ti GPUs, CUDA Version: 10.1, NVIDIA driver version: 418.56.<br><br>Tree models were trained using Python 2.7.5, XGBoost 0.81.

For manuscripts utilizing custom algorithms or software that are central to the research but not yet described in published literature, software must be made available to editors and reviewers. We strongly encourage code deposition in a community repository (e.g. GitHub). See the Nature Portfolio guidelines for submitting code & software for further information.

Data

Policy information about availability of data

All manuscripts must include a data availability statement. This statement should provide the following information, where applicable:

- Accession codes, unique identifiers, or web links for publicly available datasets
- A description of any restrictions on data availability
- For clinical datasets or third party data, please ensure that the statement adheres to our policy

The OR datasets generated during and/or analyzed during the current study are not publicly available due to institutional restrictions on data sharing and privacy concerns. The MIMIC-III dataset is publicly available: https://mimic.physionet.org/ [20].

[20] Johnson, A. E. et al. MIMIC-III, a freely accessible critical care database. Scientific Data 3, 160035 (2016).

Field-specific reporting

Please select the one below that is the best fit for your research. If you are not sure, read the appropriate sections before making your selection.

☒ Life sciences ☐ Behavioural & social sciences ☐ Ecological, evolutionary & environmental sciences

For a reference copy of the document with all sections, see nature.com/documents/nr-reporting-summary-flat.pdf

Life sciences study design

All studies must disclose on these points even when the disclosure is negative.

Sample size	Sample size calculations were unnecessary for our analysis, because we do not perform statistical tests. We do however, report the sizes of the final data matrices we use to evaluate our predictive models for each outcome and operating room data set:<br>Hypoxemia, n (OR0)=3920564, n (OR1)=4167959<br>Hypocapnia, n (OR0)=1259768, n (OR1)=1754091<br>Hypotension, n (OR0)=1837676, n (OR1)=2332902<br>Hypertension, n (OR0)=2470632, n (OR1)=2534954<br>Phenylephrine, n (OR0)=2690484, n (OR1)=2004857<br>Epinephrine, n (OR0)=97627, n (OR1)=30916<br><br>The number of procedures for each data set is: n (OR0)=29035, n (OR1)=28136, n (ICU)=1669
Data exclusions	For hypoxemia, hypocapnia, hypotension, and hypertension outcomes we filter samples that are currently hypoxemic, hypocapnic, hypotensive, or hypertensive respectively. This is to make our classification objective "forecasting new adverse events", rather than "forecasting averse events".<br><br>For phenylephrine and epinephrine, which are much less frequently encountered we filter out procedures where phenylephrine and epinephrine were never administered. This is because there would be far too many negative samples otherwise, so we focus on forecasting phenylephrine and epinephrine only in surgeries where they were administered.
Replication	In terms of replication, we replicate our experiments across six distinct outcomes: hypoxemia, hypocapnia, hypotension, hypertension, phenylephrine, and epinephrine.
Randomization	We allocate into train, validation, and test sets. We randomly split according to procedures in order to partition the test set. Splitting by procedures ensures that our models do not unfairly know about the final procedures in the final test evaluation Then, to split the validation set from the train_validation set, we split according to sample sizes.
Blinding	Blinding to group allocation is typically relevant to randomized controlled trials which focus on inference. Instead, our study focuses on the goal of prediction and the relevant randomization can be performed retrospectively.

Reporting for specific materials, systems and methods

We require information from authors about some types of materials, experimental systems and methods used in many studies. Here, indicate whether each material, system or method listed is relevant to your study. If you are not sure if a list item applies to your research, read the appropriate section before selecting a response.

Materials & experimental systems

n/a	Involved in the study
☒	☐ Antibodies
☒	☐ Eukaryotic cell lines
☒	☐ Palaeontology and archaeology
☒	☐ Animals and other organisms
☐	☒ Human research participants
☒	☐ Clinical data
☒	☐ Dual use research of concern

Methods

n/a	Involved in the study
☒	☐ ChIP-seq
☒	☐ Flow cytometry
☒	☐ MRI-based neuroimaging

Human research participants

Policy information about studies involving human research participants

Population characteristics	The characteristics for each of our three populations are included in Table 1 of the manuscript and in more detail in Supplementary Section 1.1.3.
Recruitment	The OR data is automatically collected by anesthesia monitoring systems and is not subject to self-selection biases.
Ethics oversight	The electronic data for the operating room study data was retrieved from institutional electronic medical record and data warehouse systems after receiving approval from the Institutional Review Board (University of Washington Human Subjects Division, Approval no. 46889). Protected health information was excluded from the dataset that was used for the machine-learning methods.<br><br>The electronic data for the intensive care unit study data was retrieved from the PhysioNet Clinical Databases after data use agreement approval.

Note that full information on the approval of the study protocol must also be provided in the manuscript.
